# Association between dietary phosphorus intake and chronic constipation in adults: evidence from the National Health and Nutrition Examination Survey

**DOI:** 10.1186/s12876-022-02629-8

**Published:** 2023-01-24

**Authors:** Xuelian Zhao, Lizhu Wang, Longfang Quan

**Affiliations:** 1 The First Clinical College, Shandong University of Traditional Chinese Medicine, Jinan, 250013 Shandong Province People’s Republic of China; 2 Department of Anorectal, Affiliated Hospital of Shandong University of Traditional Chinese Medicine, Jinan, 250014 Shandong Province People’s Republic of China; 3 Department of Anorectal, China Academy of Chinese Medical Sciences Xiyuan Hospital, Beijing, 100091 People’s Republic of China

**Keywords:** Phosphorus, Chronic constipation, National Health and Nutrition Examination Survey, Bowel health, Stool frequency, Stool consistency

## Abstract

**Background:**

Several studies suggest a link between micronutrients and constipation. However, the relationship between constipation and phosphorus has rarely been examined. The main aim of this study was to investigate the association between changes in the prevalence of chronic constipation and dietary phosphorus intake among adult respondents of the National Health and Nutritional Examination Survey (NHANES).

**Methods:**

Data were extracted from the NHANES database for the years 2005–2010. A total of 13,948 people were included in the analysis. Dietary information was collected using the respondents’ 24-h dietary records. We conducted multiple logistic regression analyses to examine the correlation between phosphorus intake and poor bowel movement. The primary and secondary outcomes was constipation defined by stool consistency and stool frequency, respectively.

**Results:**

Following multi-variate adjustment in model III, a significant association between chronic constipation and each additional 0.1-g intake of dietary phosphorus (odds ratio [OR], 0.97; 95% confidence interval [CI], 0.95, 1.00; *P* = 0.034 for stool consistency vs. OR, 0.94; 95% CI, 0.90, 0.99; *P* = 0.027 for stool frequency) was observed. Following multi-variate adjustment in model III, OR values and 95% CI from the second to fourth quartiles compared to the first quartile (reference group) were 0.92 (0.66, 1.27), 0.73 (0.47, 1.13), and 0.39 (0.20, 0.76), respectively, using the stool frequency definition.

**Conclusions:**

This study revealed a negative correlation between phosphorus intake and chronic constipation. This may be due to the fact that dietary phosphorus intake is associated with softer stools and increased stool frequency. Further studies in different settings should be considered to verify these findings.

**Supplementary Information:**

The online version contains supplementary material available at 10.1186/s12876-022-02629-8.

## Background

Constipation describes a symptom of difficulty passing stool. It can manifest as infrequent stools, hard stools, excessive tension, a feeling of incomplete bowel movements, and even the need to assist with defecation by hand [1]. In a meta-analysis integrating 100 papers, the combined prevalence of chronic constipation was 14% (95% confidence interval [CI]: 12–17%) [2]. The incidence of chronic constipation varies according to sex, and women are approximately twice as likely to suffer from constipation than do men [2]. In addition, constipation is affected by factors such as age and socioeconomic status [3]. The preferred treatment option for people with chronic constipation is usually diet and lifestyle regulation [3].

Phosphorus is an indispensable trace element in the human body [4]. Phosphate enemas are used to treat constipation because of their permeability, which increases stool bulk [5]. Sodium phosphate enema therapy is effective in the treatment of chronic constipation [6]. Nevertheless, the relationship between the prevalence of chronic constipation and changes in dietary phosphorus intake has been poorly studied in the general population. Although a relationship between the intake of trace elements and chronic constipation has been reported, few studies have focused on the relationship between the changes in phosphorus intake among trace elements and chronic constipation [7]. Phosphorus is found in many foods, including meat and poultry, with the highest concentration in milk and its processed products [8].

In studies published using the National Health and Nutritional Examination Survey (NHANES) database, constipation is generally defined by stool frequency and consistency [9, 10]. Different prevalence rates of constipation have been reported according to the definition used [3, 7]. Therefore, whether differences in the definition of constipation affect the relationship between the prevalence of chronic constipation and changes in dietary phosphorus intake remains unknown.

The NHANES database is a large population-based database based in the United States. We used the NHANES database to statistically control for relevant confounding factors and explore the relationship between the intake of the trace element phosphorus and chronic constipation. This study provides a reference for future research on the relationship between the dietary intake of phosphorus and chronic constipation.

## Methods

### Study participants

We extracted data on respondents from the NHANES database of cross-sectional studies for the years 2005–2010 divided into three 2-yearly cycles (2005–2006, 2007–2008, and 2009–2010). The data for these three cycles included a bowel health questionnaire (BHQ). These data were collected using a hierarchical multilevel probability design, allowing for a weighted analysis of the population. The NHANES data collection was approved by the National Center for Health Statistics’ Ethics Review Board. Each participant signed an informed consent form. The NHANES database is organized by the Center for Disease Control and Prevention, National Center for Health Statistics (Atlanta, GA, USA).

**Fig. 1:**
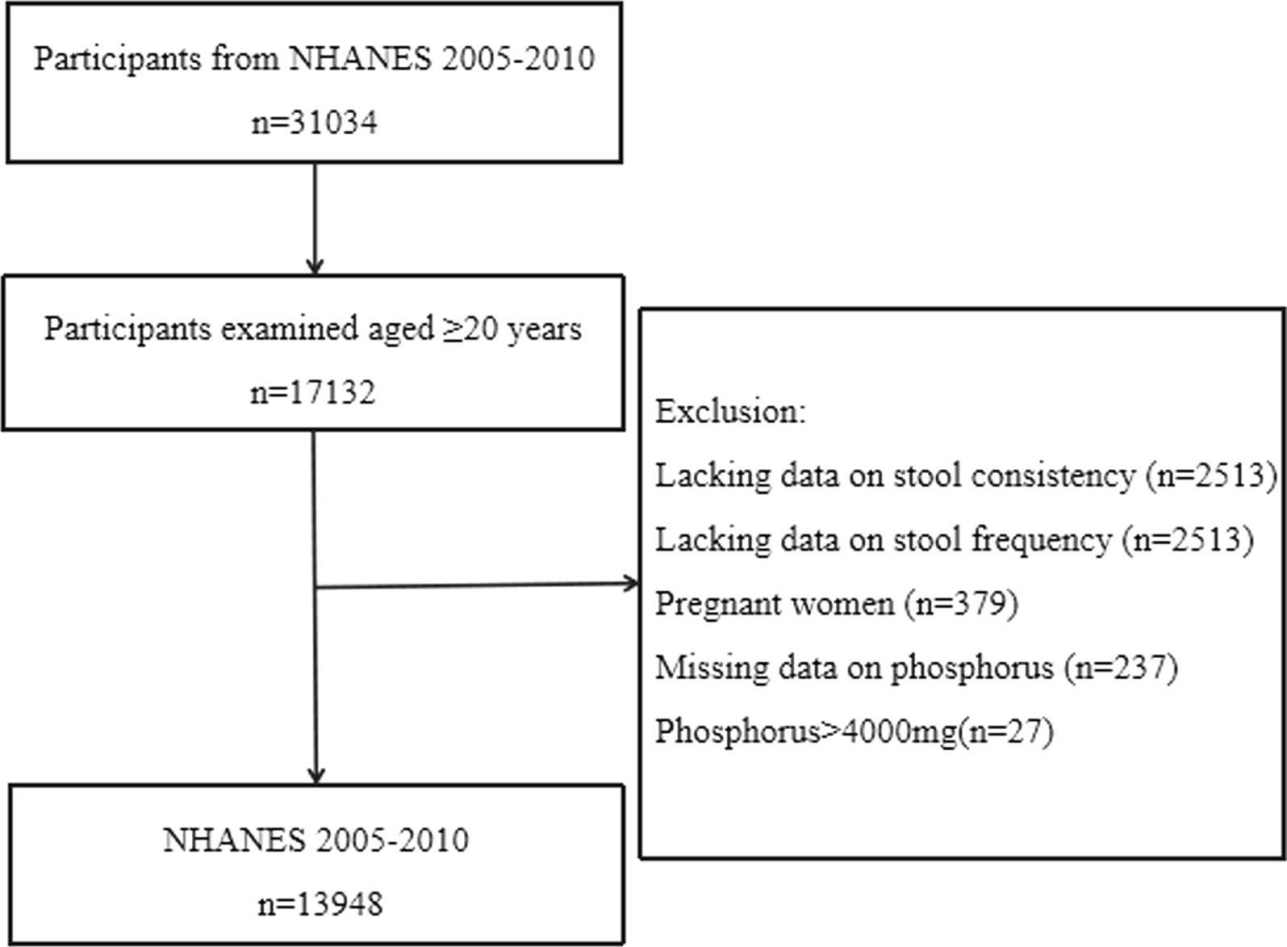
A flow chart of the process. *Notes* A flow chart of the process for the selection of eligible participants using data from the National Health and Nutrition Examination Survey (NHANES) 2005–2010

Data from 17,132 individuals aged ≥ 20 years were collected from the 2005–2010 NHANES database. Data for 2541 individuals lacking information on stool consistency and frequency were removed. Moreover, 643 participants were excluded from the study due to the following reasons: lack of data on dietary phosphorus intake (237), pregnancy (379), and phosphorus levels > 4000 mg (27). The detailed flow chart of population selection is presented in Fig. 1.

### Definition of constipation

We defined chronic constipation using the description of stool frequency and consistency in the detailed BHQ used in the 2005–2006, 2007–2008, and 2009–2010 cycles. Previous studies have shown that faecal traits can predict intestinal passage time [11] and have used stool traits to define constipation [9, 12]. The Bristol Stool Form Scale classifies stool consistency into seven types. The first two categories (Type 1: separate hard lumps, such as nuts; Type 2: sausage-like, but lumpy) were regarded as constipation, while the remaining types were considered as non-constipation.

The outcome of this study was constipation. Specifically, the primary and secondary outcome was constipation defined by stool consistency stool frequency, respectively.

When collecting information on stool frequency, participants were asked the following question: “How many times a week do you usually have a bowel movement?” Constipation was defined as a stool frequency not exceeding two bowel movements per week, and non-constipation was defined as a stool frequency of over two bowel movements per week.

A sensitivity analysis was implemented using self-reported constipation, laxative use status, and the frequency of laxative use. In self-reported constipation, non-constipation and constipation were classified according to self-reported constipation questionnaires (BHQ080). Laxative use was categorized as “used” and “not used” (BHQ 100). As for the frequency of laxative use, participants were asked “how many times have you taken laxatives or stool softeners in the past 30 days?” (BHQ110). If respondents answered that they used laxative on most days, use was defined as frequent. Use of laxatives 2–3 times per month, once per month, or 1–3 times per month was described as infrequent. As the three-constipation-related symptoms were only used in the 2009–2010 cycle, we performed the sensitivity analysis for this cycle only [13].

### Phosphorus intake

The collection of dietary information has been previously described [12, 14, 15]. The multi-pass approach was used to collect phosphorus intake data from 24-h dietary recall. Accurate information on food and drink intake was collected during the 24-h period. Each participant was interviewed twice, and dietary recalls were collected on both occasions. The first recall was based on a face-to-face interview with investigators at the Mobile Examination Center, and the second recall was conducted by telephone interview 3–10 days later. We used the mean phosphorus intake from the two dietary recalls for the analysis, and if the participants had information missing from the second dietary interview, we used the information from the first interview.

### Covariates

In accordance with previous reports on phosphorus and gut-related diseases, we included the following relevant covariates: milk consumption and energy, total fat, dietary fibre, selenium, magnesium, calcium, sodium, potassium, plain water, tap water, bottled water, tea, and coffee intake, smoking and drinking status, diagnosis of diabetes, hypertension, or depression or presence of poor oral health; income-poverty ratio, physical activity, body mass index (BMI), education, age, sex, and ethnicity [14–16]. We divided participants according to three age groups (< 45, ≥ 45 and < 65, and ≥ 65 years old). The following race and ethnicity categories were used: Non-Hispanic Black, Non-Hispanic White, Mexican American, Other Hispanic, and Other. Education level was divided into the following categories: < high school, high school, and > high school. The family income-to-poverty ratio (%) is the ratio of family income to the federal poverty threshold and the cut-off points were < 2 and ≥ 2. According to the classification of previous literature [13], we used the weekly metabolic equivalent of task (MET) minutes to classify participants based on physical activity as inactive (< 500 MET-min/week) or active (≥ 500 MET-min/week). We categorized participants according to smoking status as follows: never smokers (participants who had smoked < 100 cigarettes in their lifetime), former smokers (those who had smoked > 100 cigarettes in their lifetime but did not currently smoke), and now smokers (those who had smoked > 100 cigarettes in their lifetime and still smoked on some days or every day). Participants were categorized as drinkers if they drank ≥ 12 alcoholic beverages per year, otherwise non-drinkers. Regarding the frequency of milk consumption, participants were categorized as never drinking, rarely drinking (less than a week), sometimes drinking (once a week or more, but less than once a day), and frequently drinking (once a day or more). Based on BMI (kg/m^2^), participants were divided into three categories: obese (≥ 30), overweight (25–29.9), and under/normal weight (< 25). A diagnosis of diabetes was made if any of the following criteria applied: (1) a diagnosis of diabetes mellitus by a doctor; (2) a plasma glucose level taken 2 h after an oral glucose tolerance test or a random blood glucose level ≥ 11.1 mmol/L; (3) a fasting blood glucose ≥ 7.0 mmol/L; (4) glycohemoglobin HbA1 ≥ 6.5%; (5) participants undergoing treatment with oral medication for diabetes or intramuscular insulin. We used hypertension medication use, hypertension-related questionnaires, and systolic and diastolic blood pressure readings to determine whether the participant had hypertension. Participants with a Patient Health Questionnaire (PHQ-9) score ≥ 10 were diagnosed with depression. Interviewers collected dietary information regarding the following: total fibre (tertile 1: T1 < 11.6; tertile 2: T2, 11.6–18.0; tertile 2: T3 ≥ 18.1 g/day), total fat (T1 < 55.4; T2, 55.4–85.0; T3 ≥ 85.1 g/day), protein (T1 < 61.0; T2, 61.1–88.1; T3 ≥ 88.2 g/day), calcium (T1 < 644.5; T2, 644.5–1004.0; T3 ≥ 1004.1 mg/day), sodium (T1 < 2523.5; T2, 2523.5–3689.0; T3 ≥ 3 689.1 mg/day), potassium (T1 < 2060.0; T2, 2060–2910.0; T3 ≥ 2910.1 mg/day), total energy (T1 < 1590.0; T2, 1590.0–2247.0; T3 ≥ 2247.1 kcal/day); intake of plain water (T1 < 385.1; T2, 385.1–1059.1; T3 ≥ 1059.1 g/day; missing data), tap water (= 0; > 0 g/day; missing data), bottled water (= 0; > 0 g/day; missing data), tea (= 0; > 0 g/day; missing data), and coffee (= 0; > 0 g/day; missing data).

### Statistical analyses

We explored the relationship between changes in dietary phosphorus intake and the prevalence of chronic constipation in the NHANES dataset using statistical methods for weighted sampling. As oversampling had occurred in some populations, the weighted statistical method was applied to the data to avoid bias and ensure accuracy of the results. We set the continuous variables as categorical variables based on a previous study [13]. Categorical variables are expressed as weighted percentages and confidence intervals (95% CI). Furthermore, we investigated the relationship between changes in dietary phosphorus intake and the prevalence of chronic constipation using several multiple logistic regression models adjusted for relevant confounders. Model I is the unadjusted model. In model II, the data were adjusted for participant age (< 45; ≥ 45 < 65; ≥ 65 years old), sex, and ethnicity (Non-Hispanic White, Non-Hispanic Black, Mexican American, Other Hispanic, or Other). Model-III was further adjusted for income-poverty ratio (%) (< 2, ≥ 2, or missing data), Physical activity (MET-min/week) (< 500, ≥ 500, or missing data), BMI (kg/m^2^) (< 25, 25–29.9, or ≥ 30), poor oral health (yes, no, or missing data), hypertension (yes or no), depression (yes or no), diabetes (yes or no), smoking status (never, former, or now), drinking status (yes or no), milk consumption (often, sometimes, rarely, or never), and energy (T1 < 1590.0; T2, 1590.0–2247.0, T3 ≥ 2247.1 kcal/day).

In addition, we used smooth curve fitting after adjusting for confounding factors to show more intuitively the relationship between the changes in dietary phosphorus intake and the prevalence of chronic constipation. In the curve fitting graph, the middle line represents the effect size, and the area on both sides of the line represents the 95% CI. We further applied interaction, stratified analysis, and univariate analysis based on the variables, including age (< 45, ≥ 45 < 65, or ≥ 65 years old), sex, and ethnicity (Non-Hispanic White, Non-Hispanic Black, Mexican American, Other Hispanic, or Other), BMI (kg/m^2^) (< 25, 25–29.9, or ≥ 30), Physical activity (MET-min/week) (< 500, ≥ 500, or missing data), poor oral health (yes, no, or missing data), hypertension (yes or no), depression (yes or no), diabetes (yes or no), smoking status (never, former, or now), drinking status (yes or no), milk consumption (often, sometimes, rarely, or never), plain water (T1 < 385.1; T2, 385.1–1059.1; T3 ≥ 1059.1 g/day; missing data), tap water (= 0; > 0 g/day; missing data), bottled water (= 0; > 0 g/day; missing data), tea (= 0; > 0 g/day; missing data), coffee (= 0; > 0 g/day; missing data), energy (T1 < 1590.0; T2, 1590.0–2247.0; T3 ≥ 2247.1 kcal/day), and income-poverty ratio (%) (< 2, ≥ 2, or missing data). Due to the missing data on income-poverty ratio (%), physical activity, poor oral health, and intake of plain water, tap water, bottled water, tea, and coffee, we categorized participants with missing data as a separate group for analysis [17]. We used multiple imputation based on five replications and a chained equation approach method in the R MI procedure, to account for the missing data.

All statistical analyses were performed using R packages (The R Foundation; http://www.r-project.org; version 3.4.3) and Empower (R) (www.empowerstats. com, X&Y solutions, inc. Boston, Massachusetts). A *P* value < 0.05 was considered statistically significant.

## Results

### Clinical characteristics

Using the Bristol Stool Form Scale to define chronic constipation, the weighted prevalence of chronic constipation was 6.9% (95% CI, 6.4–7.4%) in the United States; when we defined constipation by stool frequency, the prevalence of constipation was 3.3% (95% CI, 2.8–3.9%) in the United States. Table 1 and Additional file 1: Table S2 show the basic characteristics of the population with constipation defined by the Bristol Stool Form Scale and stool frequency, respectively. Chronic constipation is associated with sex; ethnicity; BMI; education; income-poverty ratio; poor oral health; physical activity; depression; smoking status; drinking status; milk consumption; dietary phosphorus intake; and plain water, tap water, and coffee intakes (*P* < 0.05) (Table 1). However, there was no association with age, hypertension, diabetes, and intake of bottled water and tea. Certain differences were observed between results obtained using a definition of constipation based on stool consistency and those defined using stool frequency; of which, the difference in age was significant, while no significant difference was found for BMI and physical activity (Additional file 1: Table S2).

**Table 1:** Baseline Characteristics of the Study Population from National Health and Nutrition Examination Survey 2005–2010 (Using the Stool Consistency Definition of Constipation), weighted

Characteristic	No constipation (unweight n = 12,899; weight n = 181,298,423)			Constipation (unweight n = 1049; weight n = 13,397,989)			*P* value
	n	Proportion % (95% CI)	SE of %	n	Proportion % (95% CI)	SE of %	
*Sex*							< 0.001
Female	6214	49.7 (48.6,50.7)	0.5	706	71.4 (67.4,75.1)	1.9	
Male	6685	50.3 (49.3,51.4)	0.5	343	28.6 (24.9,32.6)	1.9	
*Age (yr)*							0.105
< 45	5301	45.8 (43.7,47.9)	1.0	479	49.8 (45.8,53.8)	2.0	
≥ 45, < 65	4423	36.9 (35.6,38.3)	0.7	342	32.7 (29.0,36.6)	1.9	
≥ 65	3175	17.3 (16.0,18.6)	0.6	246	17.5 (15.0,20.4)	1.3	
*Ethnicity*							< 0.001
Non-Hispanic White	6465	72.4 (68.6,75.9)	1.8	456	64.2 (57.2,70.7)	3.4	
Mexican American	2309	7.8 (6.1,9.8)	0.9	191	9.7 (7.3,12.7)	1.3	
Non-Hispanic Black	2542	10.7 (9.0,12.6)	0.9	245	15.4 (11.7,19.9)	2.0	
Other Hispanic	1068	4.1 (3.1,5.5)	0.6	118	5.2 (3.6,7.4)	0.9	
Other Race	515	5.1 (4.3,5.9)	0.4	39	5.5 (3.0,9.8)	1.6	
*Education*							< 0.001
< High school	3550	17.5 (15.9,19.1)	0.8	353	24.1 (20.4,28.2)	1.9	
High School	3036	24.1 (22.7,25.5)	0.7	286	29.2 (25.4,33.4)	2.0	
> High School	6280	58.5 (56.0,60.9)	1.2	407	46.7 (41.5,51.9)	2.6	
*Income-poverty ratio (%)*							< 0.001
< 2	5393	30.3(28.4,32.3)	1.0	517	39.9 (35.5, 44.4)	2.2	
≥ 2	6578	64.2 (62.0,66.3)	1.1	451	53.2 (48.8, 57.5)	2.2	
Missing data	928	5.5 (4.8, 6.3)	0.4	81	7.0 (4.8, 9.9)	1.2	
*BMI (kg/m*^*2*^*)*							< 0.001
< 25	3589	30.9 (29.1,32.7)	0.9	364	38.4 (34.2,42.7)	2.1	
≥ 25, < 30	4417	33.6 (32.1,35.1)	0.7	342	33.9 (30.2,37.8)	1.9	
≥ 30	4782	35.5 (33.9,37.1)	0.8	331	27.8 (24.3,31.5)	1.8	
*Physical activity (MET-min/week)*							< 0.001
< 500	2405	20.3 (19.0, 21.8)	0.7	194	20.8 (17.4, 24.7)	1.8	
≥ 500	7126	59.4 (57.5, 61.3)	1.0	504	52.2 (48.6, 55.8)	1.8	
Missing data	3368	20.2 (18.9, 21.6)	0.7	351	27.0 (23.7, 30.5)	1.7	
*Poor oral health*							< 0.001
No	10,162	82.4 (81.0,83.7)	0.7	775	76.3 (72.8, 79.6)	1.7	
Yes	1680	10.0 (9.2,10.8)	0.4	170	15.7 (12.8, 19.2)	1.6	
Missing data	1057	7.6 (6.6, 8.8)	0.5	104	8.0 (6.5, 9.8)	0.8	
*Hypertension*							0.155
No	8115	67.4 (65.9,68.8)	0.7	710	69.9 (66.0,73.5)	1.9	
Yes	4782	32.6 (31.2,34.1)	0.7	339	30.1 (26.5,34.0)	1.9	
*Depression*							< 0.001
No	11,776	92.7 (91.8,93.5)	0.4	902	86.6 (83.3,89.3)	1.5	
Yes	1077	7.3 (6.5,8.2)	0.4	141	13.4 (10.7,16.7)	1.5	
*Diabetes*							0.521
No	10,579	87.4 (86.3,88.3)	0.5	877	88.2 (85.7,90.2)	1.1	
Yes	2313	12.6 (11.7,13.7)	0.5	172	11.8 (9.8,14.3)	1.1	
*Smoking status*							0.013
Never	6651	51.8 (50.0,53.6)	0.9	622	57.1 (53.2,60.9)	1.9	
Former	3349	25.2 (23.8,26.5)	0.7	221	20.8 (17.8,24.2)	1.6	
Now	2896	23.0 (21.7,24.3)	0.6	206	22.1 (18.9,25.7)	1.7	
*Drinking status*							< 0.001
No	3502	22.7 (20.8,24.7)	1.0	403	33.7 (29.9,37.7)	1.9	
Yes	9390	77.3 (75.3,79.2)	1.0	644	66.3 (62.3,70.1)	1.9	
*Milk*							0.031
Often	5222	41.8 (40.1,43.5)	0.8	460	46.0 (40.8,51.2)	2.6	
Sometimes	3657	28.7 (27.6,29.9)	0.6	257	22.8 (19.2,26.9)	1.9	
Rarely	1895	14.2 (13.3,15.2)	0.5	145	13.8 (11.0,17.1)	1.5	
Never	2077	15.3 (14.3,16.4)	0.5	181	17.4 (14.5,20.8)	1.6	
*Energy*							< 0.001
T1	4205	28.9 (27.6,30.2)	0.6	440	39.3 (34.6,44.2)	2.4	
T2	4298	34.1 (32.8,35.5)	0.7	355	35.9 (31.6,40.6)	2.3	
T3	4396	36.9 (35.3,38.6)	0.8	254	24.7 (21.9,27.8)	1.5	
*Total fat*							< 0.001
T1	4211	28.5 (27.1,29.9)	0.7	438	38.8 (34.5,43.4)	2.2	
T2	4290	33.7 (32.5,34.9)	0.6	359	35.8 (32.3,39.5)	1.8	
T3	4398	37.8 (36.2,39.5)	0.8	252	25.4 (22.3,28.6)	1.6	
*Dietary fiber*							< 0.001
T1	4175	30.1 (28.3,31.9)	0.9	447	43.5 (39.9,47.2)	1.8	
T2	4245	34.5 (33.2,35.8)	0.6	328	30.2 (26.5,34.1)	1.9	
T3	4379	35.4 (33.4,37.5)	1.0	247	26.3 (22.5,30.6)	2.0	
*Selenium*							< 0.001
T1	4192	29.6 (28.3,30.8)	0.6	455	44.0 (39.2,48.9)	2.4	
T2	4295	33.1 (31.9,34.3)	0.6	351	32.4 (28.5,36.6)	2.0	
T3	4412	37.3 (35.7,38.9)	0.8	243	23.6 (19.2,28.6)	2.3	
*Magnesium*							< 0.001
T1	4182	27.7 (26.0,29.5)	0.9	467	42.4 (38.3,46.7)	2.1	
T2	4308	33.6 (32.6,34.6)	0.5	341	33.4 (29.6,37.5)	2.0	
T3	4409	38.7 (36.9,40.5)	0.9	241	24.2 (20.6,28.2)	1.9	
*Calcium*							< 0.001
T1	4242	28.4 (26.9,30.0)	0.8	405	35.2 (31.3,39.3)	2.0	
T2	4287	33.4 (32.2,34.7)	0.6	364	36.4 (33.0,39.9)	1.7	
T3	4370	38.2 (36.4,39.9)	0.9	280	28.4 (24.6,32.5)	2.0	
*Sodium*							< 0.001
T1	4208	28.0 (26.7,29.4)	0.7	441	38.8 (34.5,43.2)	2.2	
T2	4266	33.7 (32.4,34.9)	0.6	381	38.8 (34.8,43.0)	2.1	
T3	4425	38.3 (36.9,39.7)	0.7	227	22.4 (19.9,25.2)	1.3	
*Potassium*							< 0.001
T1	4187	28.5 (26.9,30.2)	0.8	457	41.0 (37.0,45.1)	2.0	
T2	4304	32.6 (31.5,33.6)	0.5	349	32.3 (28.3,36.5)	2.0	
T3	4408	38.9 (37.1,40.7)	0.9	243	26.7 (23.3,30.4)	1.8	
*Phosphorus*							< 0.001
T1	4089	28.1 (26.5,29.7)	0.8	457	40.4 (36.7,44.2)	1.9	
T2	4311	33.7 (32.5,34.8)	0.6	340	32.8 (29.0,36.8)	1.9	
T3	4399	38.3 (36.8,39.8)	0.7	252	26.8 (23.8,30.0)	1.5	
*Plain water*							< 0.001
T1	3773	26.9 (25.4,28.6)	0.8	354	35.0 (30.3,40.1)	2.5	
T2	3796	28.9 (27.7,30.1)	0.6	334	31.4 (27.6,35.4)	2.0	
T3	3896	34.0 (32.1,35.9)	1.0	242	24.4 (21.0,28.2)	1.8	
Missing data	1434	10.2 (9.3,11.2)	0.5	119	9.2 (6.8,12.4)	1.4	
*Tap water*							0.036
= 0	4309	30.1 (28.1,32.1)	1.0	403	35.9 (30.6,41.6)	2.8	
> 0	7156	59.7 (57.5,61.9)	1.1	527	54.9 (49.9,59.8)	2.5	
Missing data	1434	10.2 (9.3,11.2)	0.5	119	9.2 (6.8,12.4)	1.4	
*Bottled water*							0.313
= 0	6179	49.8 (47.5,52.2)	1.2	510	53.3 (48.6,58.0)	2.3	
> 0	5286	40.0 (37.9,42.0)	1.0	420	37.5 (33.1,42.1)	2.3	
Missing data	1434	10.2 (9.3,11.2)	0.5	119	9.2 (6.8,12.4)	1.4	
*Coffee*							0.004
= 0	4409	35.2 (33.7,36.8)	0.8	419	42.2 (38.1,46.4)	2.1	
> 0	7055	54.6 (52.9,56.2)	0.8	510	48.6 (44.6,52.5)	2.0	
Missing data	1435	10.2 (9.3,11.2)	0.5	120	9.2 (6.8,12.4)	1.4	
*Tea*							0.659
= 0	7483	57.1 (55.2,59.0)	0.9	607	56.4 (51.0,61.6)	2.6	
> 0	3981	32.7 (30.8,34.6)	1.0	322	34.3 (29.7,39.3)	2.4	
Missing data	1435	10.2 (9.3,11.2)	0.5	120	9.2 (6.8,12.4)	1.4	

Numbers that do not add up to 100% are attributable to missing data

*BMI* body mass index, *CI* confidence interval

Table 2 presents the prevalence of chronic constipation by definition and sex. The results show differences in the prevalence of chronic constipation according to the definition used (6.9%, 95% CI 6.4, 7.4 for stool consistency vs. 3.3%, 9.5% CI 2.8, 3.9 for stool frequency). In constipation defined by stool frequency, the prevalence of chronic constipation varied according to sex (5.5%, 95% CI 4.6, 6.5 in women vs. 1.1%, 95% CI 0.8, 1.5 in men).

**Table 2:** Comparison of Constipation Prevalence According to Stool Consistency and Stool Frequency Definitions in NHANES 2005–2010, weighted

Characteristic	No constipation		Constipation	
	n	% (95% CI)	n	% (95% CI)
Stool consistency (overall)	12,899	93.1 (92.6, 93.6)	1049	6.9 (6.4, 7.4)
Female	6214	90.4 (89.6, 91.1)	706	9.6 (8.9, 10.4)
Male	6685	96.0 (95.1, 96.7)	343	4.0 (3.3, 4.9)
Stool frequency (overall)	13,463	96.7 (96.1, 97.2)	485	3.3 (2.8, 3.9)
Female	6548	95.4 (93.5, 95.4)	372	5.5 (4.6, 6.5)
Male	6915	98.9 (98.5, 99.2)	113	1.1 (0.8, 1.5)

All values are presented as percentage (95% CI)

### Dietary phosphorus intake and constipation

**Fig. 2:**
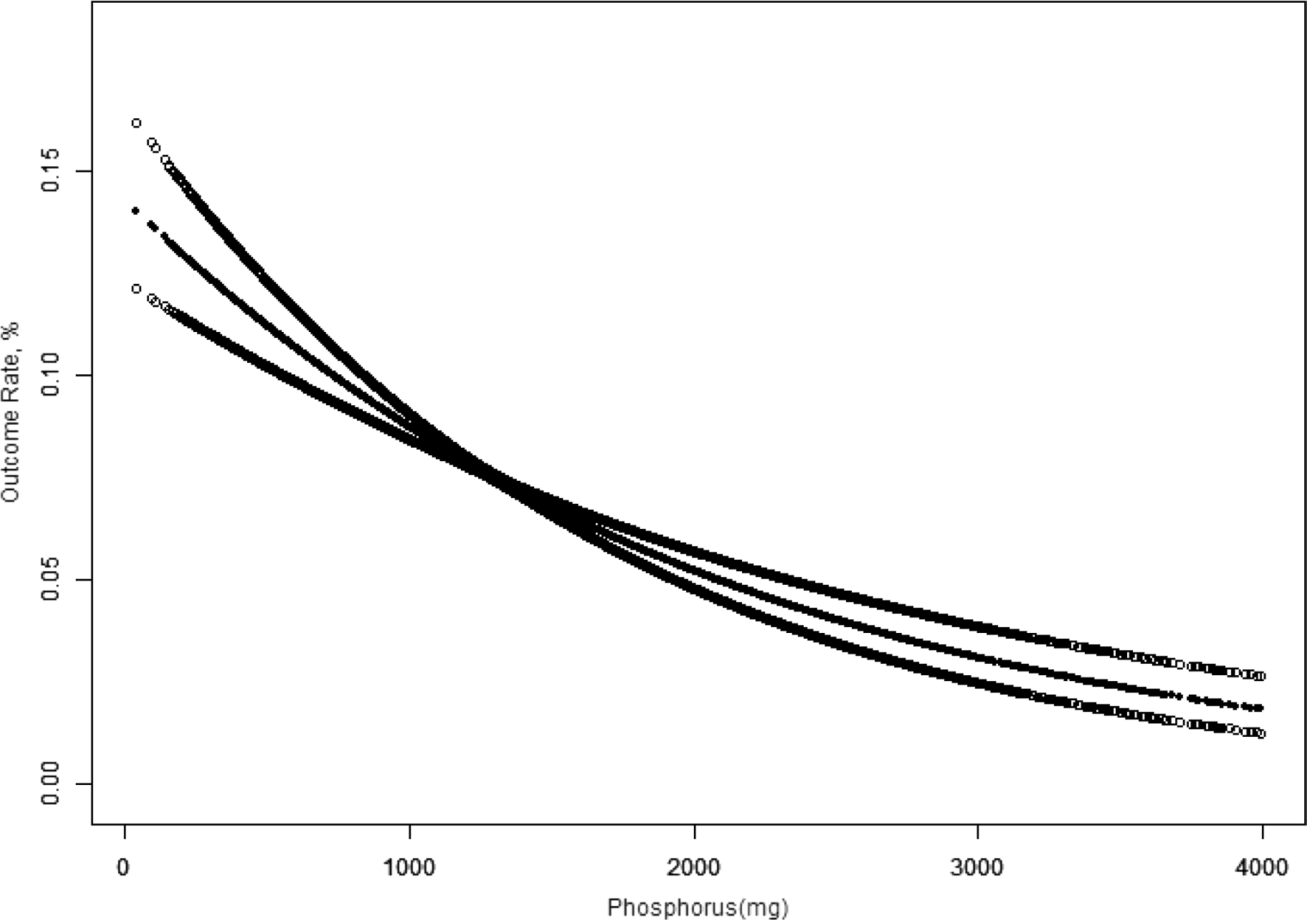
Overall Survival (Using the Stool Consistency Definition of Constipation). *Notes* the relationship between dietary phosphorus and constipation (Using the Stool Consistency Definition of Constipation), unweighted. *Adjusted for age (< 45, ≥ 45 < 65, and ≥ 65 years old), sex and ethnicity (Non-Hispanic White, Mexican American, Non-Hispanic Black, Other Hispanic, and Other Race), BMI (< 25 kg/m^2^, 25–29.9 kg/m^2^, and ≥ 30 kg/m^2^), Physical activity (MET-min/week) (< 500, ≥ 500, or missing data), poor oral health (yes, no, missing data), hypertension (yes, no), depression (yes, no), diabetes (yes, no), smoking status (never, former, and now), drinking status (yes, no), milk (often, sometimes, rarely, never), energy (T1 < 1590.0; T2, 1590.0–2247.0; T3 ≥ 2247.1 kcal/day), and income-poverty ratio (%) (< 2, ≥ 2, or missing data)

**Fig. 3:**
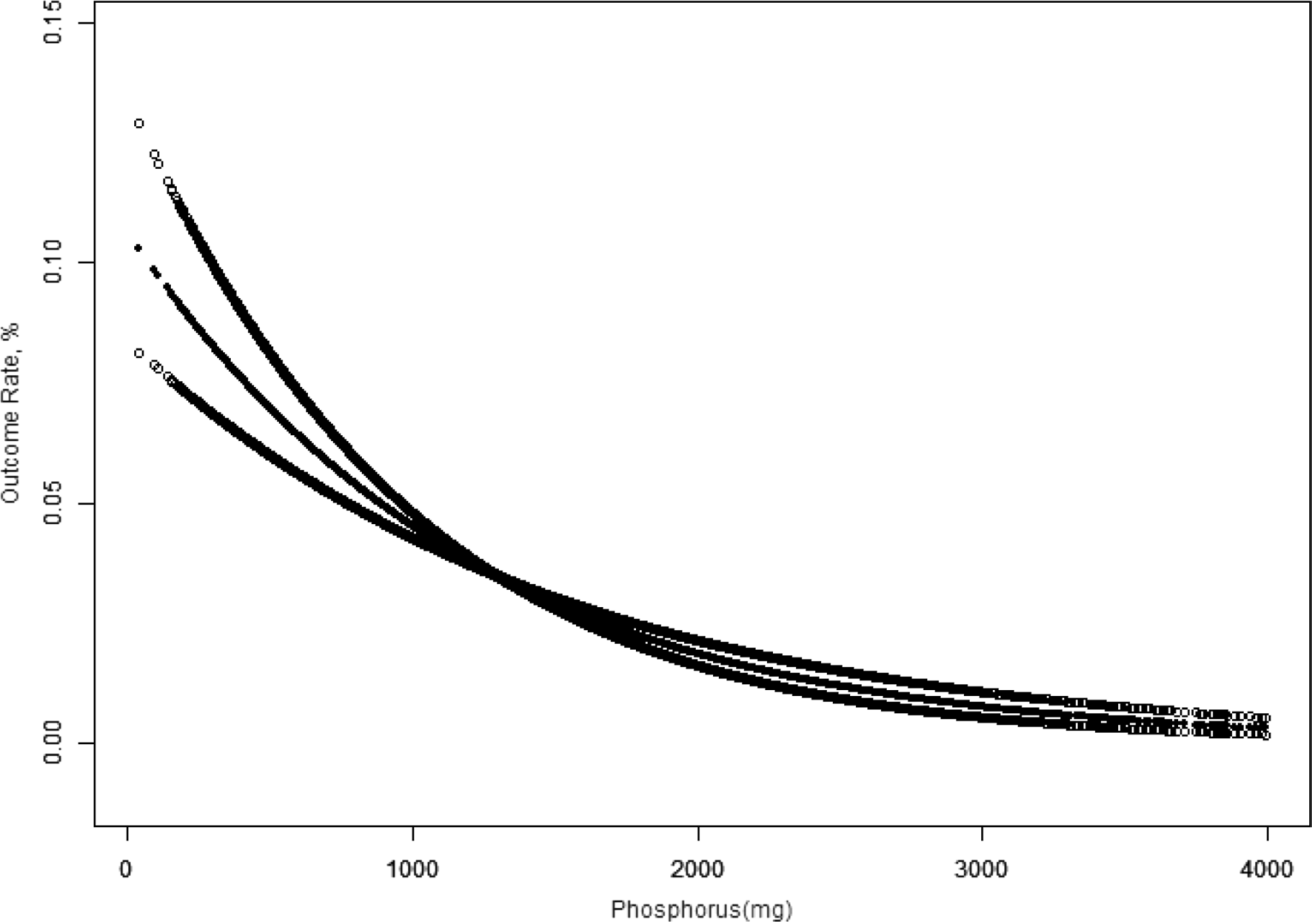
Overall Survival (Using the Stool Consistency Definition of Constipation). *Notes* the association between dietary phosphorus and constipation (Using the Stool Frequency Definition of Constipation), unweighted. *Adjusted for age (< 45, ≥ 45 < 65, and ≥ 65 years old), sex and ethnicity (Non-Hispanic White, Mexican American, Non-Hispanic Black, Other Hispanic, and Other Race), BMI (< 25 kg/m^2^, 25–29.9 kg/m^2^, and ≥ 30 kg/m^2^), Physical activity (MET-min/week) (< 500, ≥ 500, or missing data), poor oral health (yes, no, missing data), hypertension (yes, no), depression (yes, no), diabetes (yes, no), smoking status (never, former, and now), drinking status (yes, no), milk (often, sometimes, rarely, never), energy (T1 < 1590.0; T2, 1590.0–2247.0; T3 ≥ 2247.1 kcal/day), and income-poverty ratio (%) (< 2, ≥ 2, or missing data)

In Figs. 2 and 3, the fitting curve describing the relationship between dietary phosphorus intake and constipation slopes downward, indicating a negative relationship between the two. We used multiple logistic regression models to further demonstrate this relationship wherein constipation was defined by stool consistency (Table 3) and stool frequency (Table 4). The unadjusted model in Table 3 shows that dietary phosphorus intake (every additional 0.1 g) was associated with constipation, as defined by the Bristol Stool Form Scale (0.94, 0.92–0.96, *P* < 0.001). Table 4 shows a similar result for constipation defined by different bowel movements (0.89, 0.86–0.92, *P* < 0.001). Model II shows that dietary phosphorus intake remained correlated with constipation after adjusting for sex, age, and race (0.97, 0.95–0.98; *P* < 0.001 for stool consistency vs. 0.93, 0.90–0.97, *P* < 0.001 for stool frequency). Model III shows a clear association after adjusting for age (< 45, ≥ 45 < 65, and ≥ 65 years old); sex and ethnicity (Non-Hispanic White, Mexican American, Non-Hispanic Black, Other Hispanic, and Other Race); BMI (< 25 kg/m^2^, 25–29.9 kg/m^2^, and ≥ 30 kg/m^2^); Physical activity (MET-min/week) (< 500, ≥ 500, or missing data); poor oral health (yes, no, missing data); hypertension (yes, no); depression (yes, no); diabetes (yes, no); smoking status (never, former, and now); drinking status (yes, no); milk (often, sometimes, rarely, never); energy (T1 < 1590.0; T2, 1590.0–2247.0; T3 ≥ 2247.1 kcal/day); and income-poverty ratio (%) (< 2, ≥ 2, or missing data) (0.97, 0.95–1.00, P = 0.045 for stool consistency vs. 0.94, 0.90–0.99, *P* = 0.034 for stool frequency). In model III, the odds ratio (OR) (95% CI) for the second to fourth quartiles compared with the reference group were 0.92 (0.66, 1.27), 0.73 (0.47, 1.13), and 0.39 (0.20, 0.76), respectively, using the stool frequency definition (Table 4). The prevalence of constipation gradually decreased as the quartile increased (*P* for trend = 0.012). We found values for the trend test for similar trends in men (*P* = 0.032 for stool consistency vs. *P* = 0.001 for stool frequency). However, similar results were not observed in the general population defined by stool consistency (*P* for trend = 0.181), nor in women (*P* for trend = 0.413 for stool consistency vs. *P* for trend = 0.335 for stool frequency).

**Table 3:** Regression Analyses of the Association Between Dietary Phosphorus Intake and Chronic Constipation (stool consistency), weighted

Exposure	Phosphorus intake (mg)	Model I	Model II	Model III
Overall		13,948	13, 948	13, 758
Phosphorus for each 0.1 g		0.94 (0.92, 0.96)	0.97 (0.95, 0.98)	0.97 (0.95, 1.00)
Per 1 SD		0.71 (0.64, 0.78)	0.83 (0.75, 0.91)	0.86 (0.75, 0.98)
Q1	< 905.0	Ref	Ref	Ref
Q2	905.0–1215.0	0.92 (0.70, 1.20)	1.02 (0.79, 1.33)	1.10 (0.84, 1.45)
Q3	1215.5–1581.0	0.62 (0.47, 0.82)	0.77 (0.58, 1.02)	0.86 (0.65, 1.15)
Q4	> 1581.0	0.46 (0.36, 0.59)	0.70 (0.55, 0.89)	0.81 (0.56, 1.18)
p for trend		< 0.001	0.002	0.181
Men		7028	7028	6917
Phosphorus for each 0.1 g		0.95 (0.92, 0.98)	0.95 (0.92, 0.99)	0.96 (0.90, 1.02)
Per 1 SD		0.73 (0.60, 0.90)	0.75 (0.61, 0.92)	0.77 (0.54, 1.10)
Q1	< 1065.0	Ref	Ref	Ref
Q2	1065.0–1395.5	0.54 (0.35, 0.84)	0.56 (0.37,0.87)	0.63 (0.38,1.02)
Q3	1396.0–1793.5	0.59 (0.38,0.90)	0.62 (0.39,0.97)	0.67 (0.40,1.14)
Q4	> 1793.5	0.40 (0.25, 0.63)	0.42 (0.27, 0.65)	0.42 (0.20, 0.89)
p for trend		< 0.001	< 0.001	0.032
Female		6920	6920	6894
Phosphorus for each 0.1 g		0.97 (0.95, 1.00)	0.98 (0.96, 1.00)	0.98 (0.95, 1.02)
Per 1 SD		0.89 (0.81, 0.98)	0.90 (0.82, 0.99)	0.92 (0.79, 1.08)
Q1	< 803.5	Ref	Ref	Ref
Q2	803.5–1061.5	1.12 (0.83, 1.50)	1.14 (0.85, 1.54)	1.21 (0.88, 1.67)
Q3	1062.0–1348.5	0.82 (0.64, 1.07)	0.87 (0.66, 1.13)	0.96 (0.71, 1.30)
Q4	> 1348.5	0.80 (0.61, 1.06)	0.83 (0.63, 1.11)	0.93 (0.61, 1.41)
* p* for trend		0.02	0.054	0.431

Model I was not adjusted

Model II was adjusted for age, sex and ethnicity. age (< 45, ≥ 45 < 65, and ≥ 65 years old), sex and ethnicity (Non-Hispanic White, Mexican American, Non-Hispanic Black, Other Hispanic, and Other Race)

Model III was adjusted for Adjusted for age (< 45, ≥ 45 < 65, and ≥ 65 years old), sex and ethnicity (Non-Hispanic White, Mexican American, Non-Hispanic Black, Other Hispanic, and Other Race), BMI (< 25 kg/m2, 25–29.9 kg/m2, and ≥ 30 kg/m2), Physical activity (MET-min/week) (< 500, ≥ 500, or missing data), poor oral health (yes, no, missing data), hypertension (yes, no), depression (yes, no), diabetes (yes, no), smoking status (never, former, and now), drinking status (yes, no), milk (often, sometimes, rarely, never), energy (T1 < 1590.0; T2, 1590.0–2247.0; T3 ≥ 2247.1 kcal/day), and income-poverty ratio (%) (< 2, ≥ 2, or missing data)

**Table 4:** Regression Analyses of the Association Between Dietary Phosphorus Intake and Chronic Constipation (stool frequency), weighted

Exposure	Phosphorus intake (mg)	Model I	Model II	Model III
Overall		13, 948	13, 948	13, 758
Phosphorus for each 0.1 g		0.89 (0.86, 0.92)	0.93 (0.90, 0.97)	0.94 (0.90, 0.99)
Per 1 SD		0.53 (0.43, 0.64)	0.68 (0.55, 0.83)	0.72 (0.54, 0.94)
Q1	< 905.0	Ref	Ref	Ref
Q2	905.0–1215.0	0.71 (0.53, 0.95)	0.83 (0.61, 1.12)	0.92 (0.66, 1.27)
Q3	1215.5–1581.0	0.48 (0.34, 0.68)	0.65 (0.45, 0.95)	0.73 (0.47, 1.13)
Q4	> 1581.0	0.20 (0.12, 0.32)	0.36 (0.21, 0.61)	0.39 (0.20, 0.76)
P for trend		< 0.001	< 0.001	0.012
Men		7028	7028	6917
Phosphorus for each 0.1 g		0.90 (0.86, 0.95)	0.92 (0.88, 0.97)	0.92 (0.87, 0.97)
Per 1 SD		0.54 (0.40, 0.74)	0.62 (0.45, 0.004)	0.60 (0.43, 0.84)
Q1	< 1065.0	Ref	Ref	Ref
Q2	1065.0–1395.5	0.59 (0.31, 1.10)	0.68 (0.36, 1.30)	0.60 (0.27, 1.36)
Q3	1396.0–1793.5	0.26 (0.14, 0.50)	0.33 (0.17, 0.63)	0.25 (0.10, 0.62)
Q4	> 1793.5	0.22 (0.10, 0.49)	0.29 (0.13, 0.65)	0.20 (0.08, 0.50)
P for trend		< 0.001	< 0.001	0.001
Female		6920	6920	6894
Phosphorus for each 0.1 g		0.94 (0.89, 0.98)	0.94 (0.90, 0.98)	0.95 (0.88, 1.03)
Per 1 SD		0.75 (0.61, 0.93)	0.76 (0.62, 0.93)	0.81 (0.57, 1.14)
Q1	< 803.5	Ref	Ref	Ref
Q2	803.5–1061.5	0.63 (0.42, 0.94)	0.66 (0.44, 0.98)	0.74 (0.50, 1.10)
Q3	1062.0–1348.5	0.73 (0.49, 1.09)	0.77 (0.52, 1.15)	0.97 (0.67, 1.42)
Q4	> 1348.5	0.47 (0.31, 0.71)	0.47 (0.31, 0.72)	0.65 (0.37, 1.12)
P for trend		0.005	0.007	0.335

Model I was not adjusted

Model II was adjusted for age (< 45, ≥ 45 < 65, and ≥ 65 years old), sex and ethnicity (Non-Hispanic White, Mexican American, Non-Hispanic Black, Other Hispanic, and Other Race)

Model III was adjusted for Adjusted for age (< 45, ≥ 45 < 65, and ≥ 65 years old), sex and ethnicity (Non-Hispanic White, Mexican American, Non-Hispanic Black, Other Hispanic, and Other Race), BMI (< 25 kg/m2, 25–29.9 kg/m2, and ≥ 30 kg/m2), Physical activity (MET-min/week) (< 500, ≥ 500, or missing data), poor oral health (yes, no, missing data), hypertension (yes, no), depression (yes, no), diabetes (yes, no), smoking status (never, former, and now), drinking status (yes, no), milk (often, sometimes, rarely, never), energy (T1 < 1590.0; T2, 1590.0–2247.0; T3 ≥ 2247.1 kcal/day), and income-poverty ratio (%) (< 2, ≥ 2, or missing data)

To reduce the bias caused by different definitions, we performed sensitivity analysis (Additional file 1: Table S1) with three further variables that may affect the prevalence of constipation: laxative use, self-reported constipation, and the frequency of laxative use. Sensitivity analysis revealed that dietary phosphorus intake was not related to laxative use, self-reported constipation, or frequency of laxative use. Moreover, we performed a regression analysis after multiple imputation (Additional file 1: Tables S7 and S8).

### Crude association of constipation with demography, comorbidity, physical activity, smoking, alcohol consumption, and diet

Additional file 1: Table S3 for stool consistency and Additional file 1: Table S4 for stool frequency present the crude associations of constipation with demography, comorbidity, physical activity, smoking status, drinking status, and diet. Additional file 1: Table S3 shows that poor oral health, depression, and tea are associated with increased constipation. Meanwhile, the following were associated with decreased constipation: male sex, higher level of education, higher income-poverty ratio, increased BMI, higher level of physical activity, hypertension, diabetes, smoking status, drinking status, higher dietary phosphorus intake, higher plain water intake, and coffee. Additional file 1: Table S4 presents similar observed trends, except that smoking was associated with increased constipation, while often milk consumption and increased age were associated with decreased constipation.

### Stratified analysis of dietary phosphorus and chronic constipation

Additional file 1: Tables S5 and S6 show the results of stratified analysis of unadjusted variables to define constipation by the Bristol Stool Form Scale and different bowel movements, showing the relationship between dietary phosphorus intake and chronic constipation in different layers. Stratification was based on the relevant covariates. The relationship between dietary phosphorus intake and chronic constipation showed a decreasing trend, despite the application of two different definitions of chronic constipation. In Additional file 1: Table S5, significant interactions were observed when stratified by hypertension (*P* = 0.022), smoking (*P* = 0.028), income-poverty ratio (*P* = 0.042), and drinking (*P* = 0.036). In Additional file 1: Table S6, significant interactions were observed when stratified by depression (*P* = 0.038) and intake of plain water (*P* = 0.004), tap water (*P* = 0.003), bottled water (*P* = 0.002), tea (*P* = 0.002), and coffee (*P* < 0.001).

## Discussion

We screened data from the 2005 to 2010 NHANES database to investigate whether dietary phosphorus intake was associated with chronic constipation in people aged > 20 years. After controlling for other relevant confounding factors, increased dietary phosphorus intake still relieved chronic constipation. The commonly used diagnostic criteria for constipation are the Rome IV criteria, which classify constipation as functional constipation, opioid-induced constipation, functional defecation disorder, and the irritable bowel syndrome with constipation [1]. In an epidemiological survey conducted in the UK, US, and Canada, the prevalence of functional constipation according to the Rome IV criteria was at 7.9–8.6% [18].

Our definition of chronic constipation was based on stool frequency and consistency, which were derived from the number of bowel movements and the Bristol Stool Form Scale, respectively [19]. In calculating the prevalence of chronic constipation, constipation defined by stool frequency was lower than that defined by stool consistency (3.3%, 9.5% CI 2.8, 3.9 vs. 6.9%, 95% CI 6.4, 7.4, respectively). This is consistent with previously reported findings [12, 20].

Sources of dietary phosphorus include meat, dairy products, and grain products (particularly, bread and non-diary snacks and sweets), with grain products being the most abundant dietary source [21]. Some studies have shown that bread can alleviate symptoms of chronic constipation [22, 23]. A small case–control study of 46 patients found that in the colestilan group, wherein patients were orally administered with colestilan, 2 g, three times a day for two weeks, a phosphate binder, serum phosphorus was reduced and gastrointestinal symptoms, such as constipation, were exacerbated [24]. Another study found that girls with inflammatory bowel disease (IBD) consumed significantly higher amounts of phosphorus than did girls in the control group [25]. Furthermore, several studies showed that the main adverse effect of lanthanum carbonate, an oral phosphate binder, was gastrointestinal and included severe constipation [26–28]. Sodium phosphate enemas are widely used to treat constipation and are rarely associated with side effects [5]. Sodium phosphate is a hyperosmotic agent [29] and can use high osmotic pressure to form osmotic diarrhoea [30]. Moreover, it can absorb water into the intestinal tract [29]. This increases the volume of faeces, causing expansion of the intestinal lumen, promoting intestinal tract peristalsis and excretion of the intestinal contents, thereby relieving constipation [31]. A study in pigs showed that phosphorus intake may affect gut microbial composition and activity [32]. Furthermore, intestinal flora is reportedly affected by constipation [33]. Therefore, we postulate that phosphorus intake may affect constipation by affecting the intestinal flora. Additionally, lack of phosphorus intake in the diet of ducks has been shown to impair the digestion and absorption functions of the intestinal tract [34], which may lead to constipation. Nevertheless, few studies have quantified dietary phosphorus intake and examined its association with chronic constipation. Phosphorus is an indispensable trace element and participates in several physiological activities [8]. Moreover, constipation is a common disease worldwide [3]. Several studies have examined the association but failed to qualify the intake of dietary phosphorous. Therefore, further studies are essential to examine the relationship between constipation and phosphorus. The following conditions can lead to a reduction in phosphorus levels in the body: reduced oral intake, excess loss due to diarrhoea, reduced intake due to poor absorption, heavy alcohol consumption, and heavy use of antacids [35].

Our study showed that regardless of the type of constipation, the prevalence of chronic constipation gradually decreases with increasing dietary phosphorus intake after adjusting for relevant confounders (0.97, 0.95–1.00 for stool consistency; 0.94, 0.90–0.99 for stool frequency). We observed a non-linear relationship between dietary phosphorus intake and constipation. In patients with chronic constipation undergoing colonoscopy, the use of sodium phosphate has resulted in better bowel preparation that with the use of polyethylene glycol, demonstrating that phosphorus can relieve constipation [29]. The findings of our study also confirmed this relationship. After adjusting for relevant confounding factors, dietary phosphorus intake was negatively corrected with constipation, and the 2nd, 3rd, and 4th groups in the dietary phosphorus intake quartiles were all associated with a lower incidence of constipation. These findings were observed in both men and women, and the negative correlation was observed irrespectively of whether constipation was defined by stool frequency or stool trait. In the subgroup analysis, dietary phosphorus intake was associated with lower prevalence of constipation across all strata. This shows that the absorption rate of phosphorus is inversely related to constipation, and increased phosphorus absorption is associated with more frequent and softer bowel movements.

We identified factors associated with an increased prevalence of constipation consistent with the findings of previous cross-sectional studies [3]. Overall, we found that the following factors were associated with an increased prevalence of constipation following the stool consistency definition: female sex, lower educational status, lower BMI, low activity levels, poor oral health, no hypertension, no depression, no diabetes, never smoking, no alcohol consumption, and lower dietary phosphorus intake. We found similar trends for constipation defined by stool frequency, differing only for smoking and milk consumption. Among constipation defined by consistency, never smoker had a higher prevalence of constipation than did former and now smokers. Additionally, never, often, and varied milk drinkers had higher prevalence of constipation than did sometimes and often milk drinkers. Among constipation defined by stool frequency, now and never smokers had a higher prevalence of constipation than did former smokers, and often milk drinkers had the lowest prevalence. However, although many studies have examined the relationship between phosphorus and constipation, the results of these studies could not be aggregated due to the different age ranges of the study populations across the studies [36, 37].

There are discrepancies in the reported prevalence of chronic constipation according to sex. In one study, the prevalence of constipation was found to be up to two times higher in women, irrespective of the definition used [36]. Another study showed that men reported more frequent bowel movements than did women [38]. The results of our study also showed a noticeable difference in the prevalence of chronic constipation between men and women. Women had a higher prevalence of chronic constipation than did men, as defined by stool frequency (5.5%, 4.6–6.5 in women; 1.1%, 0.8–1.5 in men), and similar results were found for constipation defined by stool consistency (9.6%, 95% CI 8.9, 10.4 in women vs. 4.0%, 95% CI 3.3, 4.9 in men). Currently, no clear mechanism exists to explain the difference between the sexes in the prevalence of chronic constipation, and further research is required to clarify this finding [39].

This study has some limitations. First, as it was a cross-sectional study, no causal relationship could be inferred. Therefore, we cannot conclude that an increase in dietary phosphorus intake reduces the occurrence of chronic constipation. Second, the relevant information of BHQ110, BHQ100, and BHQ080 in the questionnaire only exists for the 2009–2010 cycle. Future studies should include a larger sample size. Third, the Rome IV diagnostic criteria for chronic constipation cover other content, in addition to stool frequency and consistency. Therefore, we performed a sensitivity analysis for constipation symptoms. However, we were unable to determine the real prevalence of chronic constipation in the population. Fourth, dietary data were provided by patients through recall and self-statement and recall bias could not be excluded. Moreover, the recall time interval was 24 h; therefore, the long-term eating habits of respondents were not included in the database. Fifth, our conclusions are only applicable to the population of this study and cannot be extrapolated to other populations. Nonetheless, this study has some strengths. First, the data compiled in this database is a representative sample of the entire population of the United States and contains a large amount of detailed information on participants, including their diet, lifestyle, disease, and demographic characteristics. Second, the database is generalized with caution, which further enhances its applicability. Third, in the different regression models, we adjusted for confounding factors that may have affected the results.

## Conclusions

In conclusion, increased dietary phosphorus intake was associated with decreased prevalence of chronic constipation in the population. This finding was consistent in constipation defined by both stool traits and stool frequency, after adjusting for age, sex, ethnicity, BMI, physical activity, poor oral health, hypertension, depression, diabetes, smoking status, drinking status, milk, energy, and income-poverty ratio. The improvement of constipation may be related to the fact that dietary phosphorus intake softens the stool and increases stool frequency. This finding provides suggestions for alleviating constipation symptoms. The findings of this study should be verified with prospective studies involving a more diverse population.

## Supplementary Information

**Additional file 1**.** Supplementary Table 1**. Multiple Regression of the Association Between Dietary Phosphorus and Other 3 Constipation-related Symptoms, weighted.** Supplementary Table 2**. Baseline Characteristics of the Study Population from National Health and Nutrition Examination Survey 2005-2010 (Using the Stool Frequency Definition of Constipation), weighted.** Supplementary Table 3**. Univariate Analysis of Relationship Between Phosphorus Intake with Constipation (Using the Stool Consistency Definition of Constipation).** Supplementary Table 4**. Univariate Analysis of Relationship Between Phosphorus Intake with Constipation (Using the Stool Frequency Definition of Constipation), weighted.** Supplementary Table 5**. Subgroup Analyses of the AssociationBetween Phosphorus Intake and Constipation (stool consistency), weighted.** Supplementary Table 6**. Subgroup Analyses of the Association Between Phosphorus Intake and Constipation (stool frequency), weighted.** Supplementary Table 7**. Regression Analyses of the Association Between Dietary Phosphorus Intake and Chronic Constipation (stool consistency) from Post-imputation, weighted.** Supplementary Table 8**. Regression Analyses of the Association Between Dietary Phosphorus Intake and Chronic Constipation (stool frequency) from Postimputation, weighted.

## Data Availability

The datasets used during the current study are available on the NHANES website (http://www.cdc.gov/nchs/nhanes.htm).

## Abbreviations

NHANES: National Health and Nutritional Examination Survey
CI: Confidence interval
BHQ: Bowel health questionnaire
BMI: Body mass index
IBD: Inflammatory bowel disease
OR: Odds ratio
