# Supplementary material for: Association between dietary phosphorus intake and chronic constipation in adults: evidence from the National Health and Nutrition Examination Survey
Source: BMC Gastroenterol. 2023 Jan 24;23:24. doi: 10.1186/s12876-022-02629-8 (PMC9875444; doi:10.1186/s12876-022-02629-8)
Supplement: Supplementary file 1 — Additional file 1. Supplementary Table 1. Multiple Regression of the Association Between Dietary Phosphorus and Other 3 Constipation-related Symptoms, weighted. Supplementary Table 2. Baseline Characteristics of the Study Population from National Health and Nutrition Examination Survey 2005-2010 (Using the Stool Frequency Definition of Constipation), weighted. Supplementary Table 3. Univariate Analysis of Relationship Between Phosphorus Intake with Constipation (Using the Stool Consistency Definition of Constipation). Supplementary Table 4. Univariate Analysis of Relationship Between Phosphorus Intake with Constipation (Using the Stool Frequency Definition of Constipation), weighted. Supplementary Table 5. Subgroup Analyses of the AssociationBetween Phosphorus Intake and Constipation (stool consistency), weighted. Supplementary Table 6. Subgroup Analyses of the Association Between Phosphorus Intake and Constipation (stool frequency), weighted. Supplementary Table 7. Regression Analyses of the Association Between Dietary Phosphorus Intake and Chronic Constipation (stool consistency) from Post-imputation, weighted. Supplementary Table 8. Regression Analyses of the Association Between Dietary Phosphorus Intake and Chronic Constipation (stool frequency) from Postimputation, weighted. [file 12876_2022_2629_MOESM1_ESM.docx]

**Supplementary Table 1.** Multiple Regression of the Association Between Dietary Phosphorus and Other 3 Constipation-related Symptoms, weighted

| Exposure | Model Ⅰ | Model Ⅱ | Model Ⅲ |
| --- | --- | --- | --- |
| Unweighted, n ^a^ | 5112 | 5112 | 5062 |
| Phosphorus for each 0.1g^a^ | 0.98(0.97,1.00) | 1.01(0.99,1.02) | 1.00(0.97,1.03) |
| Per 1 SD^a^ | 0.91(0.84,0.98) | 1.03(0.95,0.13) | 1.01(0.86,1.19) |
| Unweighted, n ^b^ | 5114 | 5114 | 5064 |
| Phosphorus for each 0.1g^b^ | 0.96(0.93,0.98) | 0.98(0.96,1.01) | 1.01(0.98,1.06) |
| Per 1 SD^b^ | 0.78(0.68,0.89) | 0.93(0.80,1.08) | 1.11(0.91,1.36) |
| Unweighted, n ^c^ | 572 | 572 | 558 |
| Phosphorus for each 0.1g^c^ | 1.02(0.99,1.06) | 1.02(0.98,1.07) | 1.00(0.93,1.07) |
| Per 1 SD^c^ | 1.13(0.92,1.38) | 1.14(0.87,1.48) | 1.01(0.68,1.49) |

Model Ⅰ was not adjusted

Model Ⅱ was adjusted for age (<45, ≥ 45＜65, and ≥ 65 years old), sex and ethnicity (Non-Hispanic White, Mexican American, Non-Hispanic Black, Other Hispanic, and Other Race).

Model Ⅲ was adjusted for Adjusted for age (< 45, ≥ 45＜ 65, and ≥ 65 years old), sex and ethnicity (Non-Hispanic White, Mexican American, Non-Hispanic Black, Other Hispanic, and Other Race), BMI (< 25kg/m2, 25-29.9 kg/m2, and ≥ 30 kg/m2), Physical activity (MET-min/week) (< 500, ≥ 500, or missing data), poor oral health (yes, no, missing data), hypertension (yes, no), depression (yes, no), diabetes (yes, no), smoking status (never, former, and now), drinking status (yes, no), milk (often, sometimes, rarely, never), energy (T1 < 1590.0; T2, 1590.0-2247.0; T3 ≥ 2247.1 kcal/day), and income-poverty ratio (%) (< 2, ≥ 2, or missing data).

Constipation-related symptoms are ^a^self-reported constipation, ^b^laxative use status, and ^c^the frequency of laxative use, respectively.

**Supplementary Table 2.** Baseline Characteristics of the Study Population from National Health and Nutrition Examination Survey 2005-2010 (Using the Stool Frequency Definition of Constipation), weighted.

| Characteristic | No constipation (unweight n=13463; weight n=188222079) | | | Constipation (unweight n=485; weight n=6474333) | | | P-value |
| --- | --- | --- | --- | --- | --- | --- | --- |
|  | n | Proportion % (95% CI) | SE of % | n | Proportion % (95% CI) | SE of % |  |
| Sex |  |  |  |  |  |  | <0.001 |
| Female | 6548 | 50.0 (49.1 ,51.0) | 0.5 | 372 | 84.1 (79.0 ,88.2) | 2.3 |  |
| Male | 6915 | 50.0 (49.0 ,50.9) | 0.5 | 113 | 15.9 (11.8 ,21.0) | 2.3 |  |
| Age (yr) |  |  |  |  |  |  | <0.001 |
| <45 | 5527 | 45.7 (43.7 ,47.7) | 1.0 | 253 | 57.3 (51.0 ,63.3) | 3.1 |  |
| ≥ 45,＜65 | 4606 | 36.9 (35.6 ,38.2) | 0.6 | 141 | 29.7 (23.5 ,36.7) | 3.3 |  |
| ≥ 65 | 3330 | 17.4 (16.2 ,18.7) | 0.6 | 91 | 13.1 (10.1 ,16.8) | 1.7 |  |
| Ethnicity |  |  |  |  |  |  | <0.001 |
| Non-Hispanic White | 6701 | 72.0 (68.2 ,75.6) | 1.8 | 220 | 65.2 (56.9 ,72.8) | 4 |  |
| Mexican American | 2451 | 8.0 (6.4 ,10.0) | 0.9 | 49 | 4.5 (2.6 ,7.6) | 1.2 |  |
| Non-Hispanic Black | 2607 | 10.6 (8.9 ,12.7) | 0.9 | 170 | 21.3 (16.6 ,26.9) | 2.6 |  |
| Other Hispanic | 1154 | 4.2 (3.1 ,5.6) | 0.6 | 32 | 4.1 (2.1 ,7.7) | 1.3 |  |
| Other Race | 540 | 5.1 (4.3 ,6.0) | 0.4 | 14 | 4.9 (2.2 ,10.2) | 1.8 |  |
| Education |  |  |  |  |  |  | 0.002 |
| < High school | 3751 | 17.7 (16.1 ,19.4) | 0.8 | 152 | 24.1 (19.7 ,29.1) | 2.3 |  |
| High School | 3207 | 24.3 (22.8 ,25.7) | 0.7 | 139 | 29.6 (23.2 ,37.0) | 3.4 |  |
| > High school | 6493 | 58.1 (55.5 ,60.6) | 1.3 | 194 | 46.3 (39.1 ,53.7) | 3.6 |  |
| Income-poverty ratio (%) | | | | | | | <0.001 |
| <2 | 5635 | 30.4 (28.4, 32.4) | 1.0 | 275 | 49.2 (42.3, 56.0) | 3.4 |  |
| ≥ 2 | 6856 | 64.1 (61.9, 66.1) | 1.1 | 173 | 44.7 (37.7, 51.9) | 3.6 |  |
| Missing data | 972 | 5.6 (4.8, 6.4) | 0.4 | 37 | 6.1 (3.9, 9.4) | 1.3 |  |
| BMI (kg/m^2^) |  |  |  |  |  |  | 0.077 |
| <25 | 3786 | 31.2 (29.5 ,32.9) | 0.9 | 167 | 37.5 (31.2 ,44.2) | 3.2 |  |
| ≥ 25, <30 | 4604 | 33.7 (32.3 ,35.0) | 0.7 | 155 | 32.4 (27.0 ,38.3) | 2.8 |  |
| ≥ 30 | 4956 | 35.2 (33.6 ,36.7) | 0.8 | 157 | 30.1 (24.7 ,36.2) | 2.9 |  |
| Physical activity (MET-min/week) | | |  |  |  |  | 0.203 |
| <500 | 2512 | 20.4 (19.0, 21.8) | 0.7 | 87 | 20.7 (15.5, 27.0) | 2.8 |  |
| ≥ 500 | 7408 | 59.4 (57.4, 61.3) | 1.0 | 222 | 46.4 (39.3, 53.7) | 3.6 |  |
| Missing data | 3543 | 20.3 (19.0, 21.6) | 0.7 | 176 | 32.9 (26.6, 39.9) | 3.3 |  |
| Poor oral health |  |  |  |  |  |  | <0.001 |
| No | 10593 | 82.3 (80.9, 83.6) | 0.7 | 344 | 73.0 (67.1, 78.2) | 2.8 |  |
| Yes | 1767 | 10.1 (9.3, 10.9) | 0.4 | 83 | 18..2(13.5, 24.0) | 2.6 |  |
| Missing data | 1103 | 7.6 (6.6, 8.8) | 0.5 | 58 | 8.8 (6.8, 11.4) | 1.1 |  |
| Hypertension |  |  |  |  |  |  | 0.245 |
| No | 8508 | 67.4 (65.8 ,69.0) | 0.8 | 317 | 71.0 (65.1 ,76.2) | 2.8 |  |
| Yes | 4953 | 32.6 (31.0 ,34.2) | 0.8 | 168 | 29.0 (23.8 ,34.9) | 2.8 |  |
| Depression |  |  |  |  |  |  | <0.001 |
| No | 12299 | 92.8 (91.9 ,93.6) | 0.4 | 379 | 76.8 (70.5 ,82.1) | 2.9 |  |
| Yes | 1113 | 7.2 (6.4 ,8.1) | 0.4 | 105 | 23.2 (17.9 ,29.5) | 2.9 |  |
| Diabetes |  |  |  |  |  |  | 0.256 |
| No | 11048 | 87.4 (86.3 ,88.3) | 0.5 | 408 | 89.3 (85.9 ,92.0) | 1.5 |  |
| Yes | 2408 | 12.6 (11.7 ,13.7) | 0.5 | 77 | 10.7 (8.0 ,14.1) | 1.5 |  |
| Smoking status |  |  |  |  |  |  | 0.024 |
| Never | 7017 | 52.2 (50.4 ,54.0) | 0.9 | 256 | 51.5 (44.0 ,59.0) | 3.7 |  |
| Former | 3479 | 25.1 (23.8 ,26.4) | 0.7 | 91 | 18.9 (14.5 ,24.3) | 2.4 |  |
| Now | 2964 | 22.7 (21.5 ,24.0) | 0.6 | 138 | 29.6 (22.8 ,37.4) | 3.6 |  |
| Drinking status |  |  |  |  |  |  | <0.001 |
| No | 3719 | 23.1 (21.2 ,25.0) | 0.9 | 186 | 33.7 (27.2 ,41.0) | 3.5 |  |
| Yes | 9736 | 76.9 (75.0 ,78.8) | 0.9 | 298 | 66.3 (59.0 ,72.8) | 3.5 |  |
| Milk |  |  |  |  |  |  | <0.001 |
| Often | 5522 | 42.4 (40.7 ,44.0) | 0.8 | 160 | 28.9 (24.0 ,34.3) | 2.5 |  |
| Sometimes | 3777 | 28.1 (27.0 ,29.2) | 0.6 | 137 | 32.2 (26.0 ,39.0) | 3.2 |  |
| Rarely | 1948 | 13.8 (13.0 ,14.7) | 0.4 | 92 | 22.2 (16.8 ,28.7) | 3.0 |  |
| Never | 2164 | 15.4 (14.5 ,16.4) | 0.5 | 94 | 15.9 (11.8 ,21.0) | 2.3 |  |
| Varied | 52 | 0.3 (0.2 ,0.5) | 0.1 | 2 | 0.9 (0.2 ,4.6) | 0.7 |  |
| Energy |  |  |  |  |  |  | <0.001 |
| T1 | 4438 | 29.2 (28.0 ,30.5) | 0.6 | 207 | 42.4 (37.2 ,47.8) | 2.6 |  |
| T2 | 4490 | 34.2 (32.9 ,35.5) | 0.7 | 163 | 36.4 (30.2 ,43.2) | 3.2 |  |
| T3 | 4535 | 36.6 (35.0 ,38.3) | 0.8 | 115 | 21.2 (16.0 ,27.5) | 2.9 |  |
| Total fat |  |  |  |  |  |  | <0.001 |
| T1 | 4458 | 28.7 (27.4 ,30.1) | 0.7 | 191 | 42.6 (36.6 ,48.9) | 3.1 |  |
| T2 | 4475 | 33.7 (32.6 ,34.9) | 0.6 | 174 | 36.3 (30.2 ,42.8) | 3.1 |  |
| T3 | 4530 | 37.5 (36.0 ,39.1) | 0.8 | 120 | 21.1 (16.4 ,26.7) | 2.6 |  |
| Dietary fiber |  |  |  |  |  |  | <0.001 |
| T1 | 4375 | 30.3 (28.5 ,32.1) | 0.9 | 247 | 51.6 (43.9 ,59.2) | 3.8 |  |
| T2 | 4516 | 34.3 (33.0 ,35.5) | 0.6 | 157 | 32.3 (25.4 ,40.1) | 3.7 |  |
| T3 | 4572 | 35.5 (33.5 ,37.5) | 1.0 | 81 | 16.1 (12.0 ,21.4) | 2.3 |  |
| Selenium |  |  |  |  |  |  | <0.001 |
| T1 | 4420 | 29.9 (28.7 ,31.2) | 0.6 | 227 | 49.0 (43.0 ,55.1) | 3.0 |  |
| T2 | 4490 | 33.2 (32.0 ,34.4) | 0.6 | 156 | 29.5 (24.7 ,34.7) | 2.5 |  |
| T3 | 4553 | 36.9 (35.3 ,38.5) | 0.8 | 102 | 21.5 (17.2 ,26.6) | 2.3 |  |
| Magnesium |  |  |  |  |  |  | <0.001 |
| T1 | 4384 | 27.9 (26.2 ,29.6) | 0.9 | 265 | 53.7 (46.3 ,60.9) | 3.7 |  |
| T2 | 4501 | 33.7 (32.7 ,34.8) | 0.5 | 148 | 30.3 (24.4 ,37.0) | 3.2 |  |
| T3 | 4578 | 38.4 (36.6 ,40.3) | 0.9 | 72 | 16.0 (11.7 ,21.5) | 2.4 |  |
| Calcium |  |  |  |  |  |  | <0.001 |
| T1 | 4423 | 28.3 (26.8 ,29.8) | 0.7 | 224 | 45.9 (39.9 ,52.1) | 3.1 |  |
| T2 | 4488 | 33.6 (32.4 ,34.9) | 0.6 | 163 | 33.6 (28.5 ,39.2) | 2.7 |  |
| T3 | 4552 | 38.1 (36.4 ,39.8) | 0.8 | 98 | 20.4 (16.2 ,25.4) | 2.3 |  |
| Sodium |  |  |  |  |  |  | <0.001 |
| T1 | 4433 | 28.1 (26.9 ,29.4) | 0.6 | 216 | 46.6 (40.0 ,53.3) | 3.3 |  |
| T2 | 4493 | 34.2 (33.1 ,35.3) | 0.6 | 154 | 29.6 (23.9 ,36.0) | 3.0 |  |
| T3 | 4537 | 37.7 (36.4 ,39.0) | 0.6 | 115 | 23.8 (18.4 ,30.3) | 3.0 |  |
| Potassium |  |  |  |  |  |  | <0.001 |
| T1 | 4395 | 28.7 (27.1 ,30.3) | 0.8 | 249 | 50.5 (43.4 ,57.7) | 3.6 |  |
| T2 | 4485 | 32.4 (31.4 ,33.5) | 0.5 | 168 | 35.1 (28.8 ,42.0) | 3.3 |  |
| T3 | 4583 | 38.9 (37.1 ,40.7) | 0.9 | 68 | 14.3 (10.6 ,19.1) | 2.1 |  |
| Phosphorus |  |  |  |  |  |  | <0.001 |
| T1 | 4411 | 28.3 (26.7 ,30.0) | 0.8 | 235 | 46.6 (40.6 ,52.7) | 3.0 |  |
| T2 | 4487 | 33.5 (32.3 ,34.7) | 0.6 | 164 | 36.0 (30.6 ,41.8) | 2.8 |  |
| T3 | 4565 | 38.2 (36.7 ,39.6) | 0.7 | 86 | 17.4 (13.2 ,22.7) | 2.4 |  |
| Plain water |  |  |  |  |  |  | <0.001 |
| T1 | 3932 | 27.0 (25.3 ,28.8) | 0.8 | 195 | 41.6 (34.5 ,49.0) | 2.5 |  |
| T2 | 3989 | 29.0 (27.9 ,30.2) | 0.6 | 141 | 28.7 (24.1 ,33.9) | 2.0 |  |
| T3 | 4040 | 33.7 (31.9 ,35.6) | 1.0 | 98 | 21.5 (15.4 ,29.2) | 1.8 |  |
| Missing data | 1502 | 10.2 (9.3 ,11.2) | 0.5 | 51 | 8.2 (5.7 ,11.6) | 1.4 |  |
| Tap water |  |  |  |  |  |  | <0.001 |
| =0 | 4505 | 30.0 (28.0 ,32.1) | 1.0 | 207 | 43.4 (36.1 ,51.0) | 2.8 |  |
| >0 | 7456 | 59.8 (57.5 ,62.0) | 1.1 | 227 | 48.4 (40.8 ,56.1) | 2.5 |  |
| Missing data | 1502 | 10.2 (9.3 ,11.2) | 0.5 | 51 | 8.2 (5.7 ,11.6) | 1.4 |  |
| Bottled water |  |  |  |  |  |  | 0.072 |
| =0 | 6437 | 49.8 (47.5 ,52.2) | 1.2 | 252 | 56.7 (49.9 ,63.3) | 2.3 |  |
| >0 | 5524 | 39.9 (38.0 ,42.0) | 1.0 | 182 | 35.1 (28.8 ,42.0) | 2.3 |  |
| Missing data | 1502 | 10.2 (9.3 ,11.2) | 0.5 | 51 | 8.2 (5.7 ,11.6) | 1.4 |  |
| Coffee |  |  |  |  |  |  | <0.001 |
| =0 | 4605 | 35.3 (33.8 ,36.9) | 0.8 | 223 | 46.0 (41.0 ,51.0) | 2.1 |  |
| >0 | 7354 | 54.4 (52.7 ,56.1) | 0.8 | 211 | 45.9 (41.1 ,50.8) | 2.0 |  |
| Missing data | 1504 | 10.2 (9.3 ,11.2) | 0.5 | 51 | 8.2 (5.7 ,11.6) | 1.4 |  |
| Tea |  |  |  |  |  |  | 0.405 |
| =0 | 7809 | 57.0 (55.1 ,58.9) | 0.9 | 281 | 57.4 (51.3 ,63.3) | 2.6 |  |
| >0 | 4150 | 32.7 (30.8 ,34.7) | 1.0 | 153 | 34.4 (29.6 ,39.5) | 2.4 |  |
| Missing data | 1504 | 10.2 (9.3 ,11.2) | 0.5 | 51 | 8.2 (5.7 ,11.6) | 1.4 |  |

Note：Numbers that do not add up to 100% are attributable to missing data.

BMI, body mass index, CI, confidence interval.

**Supplementary Table 3.** Univariate Analysis of Relationship Between Phosphorus Intake with Constipation (Using the Stool Consistency Definition of Constipation).

| Characteristic | n | % (95%CI) | OR (95%CI) | P-value |
| --- | --- | --- | --- | --- |
| Sex |  |  |  |  |
| Female | 6920 | 9.60 (8.89 ,10.31) | Ref. |  |
| Male | 7028 | 4.03 (3.30 ,4.76) | 0.40 (0.32, 0.48) | <0.001 |
| Age (yr) |  |  |  |  |
| <45 | 5780 | 7.44 (6.47 ,8.40) | Ref. |  |
| ≥ 45,＜65 | 4747 | 6.14 (5.25 ,7.04) | 0.82 (0.66, 1.01) | 0.070 |
| ≥ 65 | 3421 | 6.96 (6.09 ,7.84) | 0.93 (0.75, 1.15) | 0.518 |
| Ethnicity |  |  |  |  |
| Non-Hispanic White | 6921 | 6.15 (5.47 ,6.84) | Ref. |  |
| Mexican American | 2500 | 8.43 (6.48 ,10.39) | 1.40 (1.05, 1.87) | 0.025 |
| Non-Hispanic Black | 2787 | 9.64 (8.16 ,11.12) | 1.63 (1.32, 2.00) | <0.001 |
| Other Hispanic | 1186 | 8.54 (6.48 ,10.60) | 1.42 (1.07, 1.91) | 0.022 |
| Other Race | 554 | 7.43 (4.01 ,10.85) | 1.22 (0.72, 2.09) | 0.464 |
| Education |  |  |  |  |
| < High school | 3903 | 9.24 (7.90 ,10.58) | Ref. |  |
| High School | 3346 | 8.22 (7.08 ,9.37) | 0.88 (0.71, 1.09 | 0.251 |
| > High school | 6687 | 5.57 (4.96 ,6.18) | 0.58 (0.48, 0.70) | <0.001 |
| Income-poverty ratio (%) | | | | |
| <2 | 5910 | 8.86 (7.90 ,9.81) | Ref. |  |
| ≥ 2 | 7029 | 5.77 (5.08 ,6.46) | 0.63 (0.53, 0.75) | <0.001 |
| Missing data | 1009 | 8.53 (6.16, 10.91) | 0.96(0.67, 1.37) | 0.824 |
| BMI (kg/m2) |  |  |  |  |
| <25 | 3953 | 8.41 (7.17 ,9.66) | Ref. |  |
| ≥ 25, <30 | 4759 | 6.93 (5.96 ,7.91) | 0.81 (0.65, 1.02) | 0.079 |
| ≥ 30 | 5113 | 5.46 (4.79 ,6.13) | 0.63 (0.51, 0.77) | <0.001 |
| Physical activity (MET-min/week) | | | | |
| < 500 | 2599 | 7.03 (5.73, 8.33) | Ref. |  |
| ≥ 500 | 7630 | 6.09 (5.45, 6.74) | 0.86 (0.68, 1.08) | 0.196 |
| Missing data | 3719 | 8.98 (7.82, 10.14) | 1.31 (1.00, 1.71) | 0.058 |
| Poor oral health |  |  |  |  |
| No | 10937 | 6.41 (5.91 ,6.90) | Ref. |  |
| Yes | 1850 | 10.44 (8.25 ,12.64) | 1.70 (1.33, 2.17) | <0.001 |
| Missing data | 1161 | 7.15 (5.44, 8.87) | 1.13 (0.87, 1.45) | 0.366 |
| Hypertension |  |  |  |  |
| No | 8825 | 7.12 (6.48 ,7.77) | Ref. |  |
| Yes | 5121 | 6.38 (5.55 ,7.20) | 0.89 (0.75, 1.05) | 0.162 |
| Depression |  |  |  |  |
| No | 12678 | 6.45 (5.97 ,6.94) | Ref. |  |
| Yes | 1218 | 11.95 (9.03 ,14.88) | 1.97 (1.49, 2.60) | <0.001 |
| Diabetes |  |  |  |  |
| No | 11456 | 6.94 (6.35 ,7.54) | Ref. |  |
| Yes | 2485 | 6.48 (5.28 ,7.68) | 0.93 (0.74, 1.16) | 0.524 |
| Smoking status |  |  |  |  |
| Never | 7273 | 7.53 (6.91 ,8.15) | Ref. |  |
| Former | 3570 | 5.77 (4.85 ,6.68) | 0.75 (0.63, 0.90) | 0.004 |
| Now | 3102 | 6.62 (5.44 ,7.80) | 0.87 (0.72, 1.06) | 0.170 |
| Drinking status |  |  |  |  |
| No | 3905 | 9.88 (8.61 ,11.15) | Ref. |  |
| Yes | 10034 | 5.96 (5.40 ,6.52) | 0.58 (0.48, 0.69) | <0.001 |
| Milk |  |  |  |  |
| Often | 5682 | 7.50 (6.57 ,8.43) | Ref. |  |
| Sometimes | 3914 | 5.54 (4.61 ,6.46) | 0.72 (0.57, 0.92) | 0.012 |
| Rarely | 2040 | 6.68 (5.29 ,8.07) | 0.88 (0.66, 1.18) | 0.400 |
| Never | 2258 | 7.73 (6.08 ,9.39) | 1.03 (0.79, 1.35) | 0.814 |
| Varied | 54 | 12.32 (-2.69 ,27.34) | 1.73 (0.42, 7.11) | 0.450 |
| Energy |  |  |  |  |
| T1 | 4645 | 9.13 (7.89 ,10.37) | Ref. |  |
| T2 | 4653 | 7.22 (6.21 ,8.23) | 0.77 (0.60, 0.99) | 0.051 |
| T3 | 4650 | 4.71 (3.97 ,5.46) | 0.49 (0.40, 0.61) | <0.001 |
| Total fat |  |  |  |  |
| T1 | 4649 | 9.16 (8.09 ,10.23) | Ref. |  |
| T2 | 4649 | 7.28 (6.25 ,8.32) | 0.78 (0.63, 0.96) | 0.025 |
| T3 | 4650 | 4.72 (4.01 ,5.43) | 0.49 (0.39, 0.62) | <0.001 |
| Dietary fiber |  |  |  |  |
| T1 | 4622 | 9.66 (8.74 ,10.57) | Ref. |  |
| T2 | 4673 | 6.07 (5.19 ,6.96) | 0.60 (0.51, 0.71) | <0.001 |
| T3 | 4653 | 5.20 (4.43 ,5.98) | 0.51 (0.43, 0.62) | <0.001 |
| Selenium |  |  |  |  |
| T1 | 4647 | 9.91 (8.65 ,11.16) | Ref. |  |
| T2 | 4646 | 6.74 (5.92 ,7.57) | 0.66 (0.54, 0.80) | <0.001 |
| T3 | 4655 | 4.47 (3.54 ,5.39) | 0.43 (0.32, 0.56) | <0.001 |
| Magnesium |  |  |  |  |
| T1 | 4649 | 10.17 (9.06 ,11.27) | Ref. |  |
| T2 | 4649 | 6.85 (5.94 ,7.75) | 0.65 (0.54, 0.79) | <0.001 |
| T3 | 4650 | 4.41 (3.72 ,5.10) | 0.41 (0.33, 0.50) | <0.001 |
| Calcium |  |  |  |  |
| T1 | 4647 | 8.39 (7.40 ,9.38) | Ref. |  |
| T2 | 4651 | 7.45 (6.61 ,8.29) | 0.88 (0.74, 1.04) | 0.142 |
| T3 | 4650 | 5.21 (4.38 ,6.05) | 0.60 (0.48, 0.76) | <0.001 |
| Sodium |  |  |  |  |
| T1 | 4649 | 9.28 (8.12 ,10.44) | Ref. |  |
| T2 | 4647 | 7.85 (6.92 ,8.78) | 0.83 (0.67, 1.03 | 0.101 |
| T3 | 4652 | 4.14 (3.44 ,4.85) | 0.42 (0.34, 0.52 | <0.001 |
| Potassium |  |  |  |  |
| T1 | 4644 | 9.60 (8.50 ,10.69) | Ref. |  |
| T2 | 4653 | 6.83 (5.91 ,7.76) | 0.69 (0.56, 0.85) | <0.001 |
| T3 | 4651 | 4.83 (4.17 ,5.48) | 0.48 (0.40, 0.57) | <0.001 |
| Phosphorus |  |  |  |  |
| T1 | 4646 | 9.61 (8.61 ,10.61) | Ref. |  |
| T2 | 4651 | 6.72 (5.84 ,7.60) | 0.68 (0.56, 0.82) | <0.001 |
| T3 | 4651 | 4.92 (4.20 ,5.64) | 0.49 (0.41, 0.58) | <0.001 |
| Plain water |  |  |  |  |
| T1 | 4127 | 8.76 (7.46 ,10.06) | Ref. |  |
| T2 | 4130 | 7.44 (6.49 ,8.39) | 0.84 (0.67, 1.04) | 0.122 |
| T3 | 4138 | 5.04 (4.18 ,5.89) | 0.55 (0.44, 0.70) | <0.001 |
| Missing data | 1553 | 6.24 (4.39 ,8.09) | 0.69 (0.47, 1.01) | 0.064 |
| Tap water |  |  |  |  |
| =0 | 4712 | 8.11 (6.83 ,9.39) | Ref. |  |
| >0 | 7683 | 6.36 (5.76 ,6.96) | 0.77 (0.62, 0.95) | 0.018 |
| Missing data | 1553 | 6.24 (4.39 ,8.09) | 0.75 (0.50, 1.13) | 0.174 |
| Bottled water |  |  |  |  |
| =0 | 6689 | 7.33 (6.55 ,8.11) | Ref. |  |
| >0 | 5706 | 6.48 (5.65 ,7.31) | 0.88 (0.73, 1.05) | 0.166 |
| Missing data | 1553 | 6.24 (4.39 ,8.09) | 0.84 (0.61, 1.16) | 0.298 |
| Coffee |  |  |  |  |
| =0 | 4828 | 8.13 (7.21 ,9.06) | Ref. |  |
| >0 | 7565 | 6.17 (5.56 ,6.78) | 0.74 (0.64, 0.87) | <0.001 |
| Missing data | 1555 | 6.26 (4.41 ,8.12) | 0.76 (0.53, 1.08) | 0.128 |
| Tea |  |  |  |  |
| =0 | 8090 | 6.80 (6.04 ,7.56) | Ref. |  |
| >0 | 4303 | 7.21 (6.29 ,8.13) | 1.06 (0.88, 1.29) | 0.535 |
| Missing data | 1555 | 6.26 (4.41 ,8.12) | 0.92 (0.65, 1.29) | 0.618 |

**Supplementary Table 4** Univariate Analysis of Relationship Between Phosphorus Intake with Constipation (Using the Stool Frequency Definition of Constipation), weighted.

| Characteristic | n | % (95%CI) | OR (95%CI) | P-value |
| --- | --- | --- | --- | --- |
| Sex |  |  |  |  |
| Female | 6920 | 5.47 (4.56 ,6.38) | Ref. |  |
| Male | 7028 | 1.08 (0.73 ,1.44) | 0.19 (0.14, 0.27） | <0.001 |
| Age (yr) |  |  |  |  |
| <45 | 5780 | 4.13 (3.46 ,4.81) | Ref. |  |
| ≥ 45,＜65 | 4747 | 2.69 (1.83 ,3.56) | 0.64 (0.46, 0.89） | 0.011 |
| ≥ 65 | 3421 | 2.51 (1.89 ,3.14) | 0.60 (0.46, 0.79） | <0.001 |
| Ethnicity |  |  |  |  |
| Non-Hispanic White | 6921 | 3.02 (2.40 ,3.64) | Ref. |  |
| Mexican American | 2500 | 1.90 (1.25 ,2.55) | 0.62 (0.41, 0.96） | 0.036 |
| Non-Hispanic Black | 2787 | 6.45 (5.29 ,7.60) | 2.21 (1.71, 2.86） | <0.001 |
| Other Hispanic | 1186 | 3.23 (1.59 ,4.87) | 1.07 (0.59, 1.95） | 0.823 |
| Other Race | 554 | 3.18 (0.88 ,5.47) | 1.05 (0.51, 2.17） | 0.889 |
| Other Hispanic | 1186 | 3.23 (1.59 ,4.87) | 1.07 (0.59, 1.95） | 0.823 |
| Education |  |  |  |  |
| < High school | 3903 | 4.47 (3.55 ,5.40) | Ref. |  |
| High School | 3346 | 4.03 (2.94 ,5.13) | 0.90 (0.64, 1.25） | 0.524 |
| > High school | 6687 | 2.67 (2.04 ,3.31) | 0.59 (0.44, 0.78） | <0.001 |
| Income-poverty ratio (%) |  |  |  |  |
| <2 | 5910 | 5.28 (4.27 ,6.28) | Ref. |  |
| ≥ 2 | 7029 | 2.34 (1.77 ,2.92) | 0.43 (0.32, 0.58） | <0.001 |
| Missing data | 1009 | 3.63 (2.11, 5.15) | 0.68 (0.42, 1.09) | 0.114 |
| BMI (kg/m2) |  |  |  |  |
| <25 | 3953 | 3.97 (2.96 ,4.98) | Ref. | 0.154 |
| ≥ 25, <30 | 4759 | 3.20 (2.53 ,3.86) | 0.80 (0.59, 1.08） | 0.044 |
| ≥ 30 | 5113 | 2.86 (2.19 ,3.52) | 0.71 (0.52, 0.98） |  |
| Physical activity (MET-min/week) | | | | |
| < 500 | 2599 | 3.38 (2.24, 4.52) | Ref. |  |
| ≥ 500 | 7630 | 2.62 (2.09, 3.15) | 0.77 (0.51, 1.15) | 0.210 |
| Missing data | 3719 | 5.28 (3.91, 6.66) | 1.60 (1.07, 2.38) | 0.026 |
| Poor oral health |  |  |  |  |
| No | 10937 | 2.96 (2.47 ,3.46) | Ref. |  |
| Yes | 1850 | 5.83 (3.79 ,7.87) | 2.03 (1.42, 2.91） | <0.001 |
| Missing data | 1161 | 3.82 (2.79, 4.85) | 1.31 (0.96, 1.77) | 0.099 |
| Hypertension |  |  |  |  |
| No | 8825 | 3.50 (2.88 ,4.11 | Ref. |  |
| Yes | 5121 | 2.97 (2.20 ,3.74) | 0.85 (0.64, 1.12） | 0.252 |
| Depression |  |  |  |  |
| No | 12678 | 2.77 (2.32 ,3.21) | Ref. |  |
| Yes | 1218 | 9.98 (6.92 ,13.05) | 3.90 (2.76, 5.50） | <0.001 |
| Diabetes |  |  |  |  |
| No | 11456 | 3.40 (2.81 ,3.99) | Ref. |  |
| Yes | 2485 | 2.82 (2.00 ,3.65) | 0.83 (0.59, 1.15） | 0.262 |
| Smoking status |  |  |  |  |
| Never | 7273 | 3.28 (2.69 ,3.87) | Ref. |  |
| Former | 3570 | 2.53 (1.75 ,3.32) | 0.77 (0.56, 1.05） | 0.107 |
| Now | 3102 | 4.29 (3.03 ,5.54) | 1.32 (0.93, 1.86） | 0.122 |
| Drinking status |  |  |  |  |
| No | 3905 | 4.78 (3.65 ,5.92) | Ref. |  |
| Yes | 10034 | 2.87 (2.34 ,3.40) | 0.59 (0.44, 0.78） | <0.001 |
| Milk |  |  |  |  |
| Often | 5682 | 2.29 (1.88 ,2.70) | Ref. |  |
| Sometimes | 3914 | 3.79 (2.93 ,4.65) | 1.68 (1.25, 2.25) | 0.001 |
| Rarely | 2040 | 5.23 (3.49 ,6.97) | 2.35 (1.66, 3.33) | <0.001 |
| Never | 2258 | 3.42 (2.23 ,4.62) | 1.51 (1.07, 2.14) | 0.024 |
| Varied | 54 | 9.63 (-5.32 ,24.57) | 4.54 (0.79, 26.02) | 0.096 |
| Energy |  |  |  |  |
| T1 | 4645 | 4.76 (3.74 ,5.78) | Ref. |  |
| T2 | 4653 | 3.53 (2.80 ,4.27) | 0.73 (0.55, 0.97) | 0.035 |
| T3 | 4650 | 1.95 (1.29 ,2.61) | 0.40 (0.28, 0.56) | <0.001 |
| Total fat |  |  |  |  |
| T1 | 4649 | 4.86 (3.73 ,5.98) | Ref. |  |
| T2 | 4649 | 3.56 (2.84 ,4.28) | 0.72 (0.54, 0.97) | 0.037 |
| T3 | 4650 | 1.90 (1.33 ,2.47) | 0.38 (0.27, 0.53) | <0.001 |
| Dietary fiber |  |  |  |  |
| T1 | 4622 | 5.53 (4.36 ,6.71) | Ref. |  |
| T2 | 4673 | 3.14 (2.38 ,3.90) | 0.55 (0.39, 0.79) | 0.002 |
| T3 | 4653 | 1.54 (1.01 ,2.08) | 0.27 (0.18, 0.39) | <0.001 |
| Selenium |  |  |  |  |
| T1 | 4647 | 5.34 (4.27 ,6.41) | Ref. |  |
| T2 | 4646 | 2.96 (2.41 ,3.51) | 0.54 (0.42, 0.70) | <0.001 |
| T3 | 4655 | 1.97 (1.39 ,2.54) | 0.36 (0.26, 0.48) | <0.001 |
| Magnesium |  |  |  |  |
| T1 | 4649 | 6.21 (4.88 ,7.54) | Ref. |  |
| T2 | 4649 | 3.00 (2.41 ,3.59) | 0.47 (0.34, 0.65) | <0.001 |
| T3 | 4650 | 1.41 (0.89 ,1.94) | 0.22 (0.15, 0.32) | <0.001 |
| Calcium |  |  |  |  |
| T1 | 4647 | 5.29 (4.06 ,6.52) | Ref. |  |
| T2 | 4651 | 3.33 (2.74 ,3.92） | 0.62 (0.47, 0.82) | 0.002 |
| T3 | 4650 | 1.81 (1.33 ,2.30） | 0.33 (0.24, 0.45) | <0.001 |
| Sodium |  |  |  |  |
| T1 | 4649 | 5.39 (4.20 ,6.57） | Ref. |  |
| T2 | 4647 | 2.89 (2.32 ,3.47） | 0.52 (0.39, 0.71) | <0.001 |
| T3 | 4652 | 2.13 (1.47 ,2.78） | 0.38 (0.27, 0.54） | <0.001 |
| Potassium |  |  |  |  |
| T1 | 4644 | 5.72 (4.56 ,6.87） | Ref. |  |
| T2 | 4653 | 3.59 (2.87 ,4.32） | 0.61 (0.45, 0.83） | 0.003 |
| T3 | 4651 | 1.25 (0.77 ,1.73） | 0.21 (0.14, 0.31） | <0.001 |
| Phosphorus |  |  |  |  |
| T1 | 4646 | 5.35 (4.34 ,6.37） | Ref. |  |
| T2 | 4651 | 3.57 (2.80 ,4.33） | 0.65 (0.50, 0.86） | 0.004 |
| T3 | 4651 | 1.54 (1.03 ,2.05） | 0.28 (0.19, 0.39） | <0.001 |
| Plain water |  |  |  |  |
| T1 | 4127 | 5.03 (3.74 ,6.32) | Ref. |  |
| T2 | 4130 | 3.29 (2.60 ,3.99) | 0.64 (0.49, 0.85) | 0.003 |
| T3 | 4138 | 2.15 (1.48 ,2.82) | 0.41 (0.26, 0.66) | 0.001 |
| Missing data | 1553 | 2.67 (1.54 ,3.80) | 0.52 (0.33, 0.82) | 0.007 |
| Tap water |  |  |  |  |
| =0 | 4712 | 4.74 (3.65 ,5.83) | Ref. |  |
| >0 | 7683 | 2.71 (2.16 ,3.26) | 0.56 (0.41, 0.76) | 0.001 |
| Missing data | 1553 | 2.67 (1.54 ,3.80) | 0.55 (0.36, 0.85) | 0.010 |
| Bottled water |  |  |  |  |
| =0 | 6689 | 3.77 (2.98 ,4.55) | Ref. |  |
| >0 | 5706 | 2.94 (2.37 ,3.50) | 0.77 (0.59, 1.02) | 0.074 |
| Missing data | 1553 | 2.67 (1.54 ,3.80) | 0.70 (0.47, 1.05) | 0.090 |
| Coffee |  |  |  |  |
| =0 | 4828 | 4.28 (3.56 ,5.00) | Ref. |  |
| >0 | 7565 | 2.82 (2.28 ,3.36) | 0.65 (0.54, 0.79) | <0.001 |
| Missing data | 1555 | 2.67 (1.54 ,3.80) | 0.61 (0.40, 0.94) | 0.028 |
| Tea |  |  |  |  |
| =0 | 8090 | 3.35 (2.77 ,3.93) | Ref. |  |
| >0 | 4303 | 3.49 (2.77 ,4.20) | 1.04 (0.84, 1.29) | 0.696 |
| Missing data | 1555 | 2.67 (1.54 ,3.80) | 0.79 (0.52, 1.22) | 0.293 |

**Supplementary Table 5.** Subgroup Analyses of the Association Between Phosphorus Intake and Constipation (stool consistency), weighted

| Characteristic | OR (95%CI) | P for interaction |
| --- | --- | --- |
| Sex |  | 0.243 |
| Female | 0.97 (0.95, 1.00) |  |
| Male | 0.95 (0.92, 0.98) |  |
| Age (yr) |  | 0.819 |
| <45 | 0.94 (0.92, 0.96) |  |
| ≥ 45,＜65 | 0.93 (0.90, 0.97) |  |
| ≥ 65 | 0.93 (0.90, 0.97) |  |
| Ethnicity |  | 0.324 |
| Non-Hispanic White | 0.94 (0.91, 0.96) |  |
| Mexican American | 0.96 (0.93, 1.00) |  |
| Non-Hispanic Black | 0.97 (0.94, 1.00) |  |
| Other Hispanic | 0.95 (0.91, 0.99) |  |
| Other Race | 0.91 (0.80, 1.02) |  |
| Education |  | 0.750 |
| < High school | 0.94 (0.91, 0.97) |  |
| High School | 0.95 (0.92, 0.99) |  |
| > High school | 0.94 (0.92, 0.96) |  |
| Income-poverty ratio (%) |  | 0.113 |
| <2 | 0.96 (0.95, 0.98) |  |
| ≥ 2 | 0.93 (0.90, 0.96) |  |
| Missing data | 0.92 (0.86, 0.99) |  |
| BMI (kg/m2) |  | 0.847 |
| <25 | 0.94 (0.92, 0.97) |  |
| ≥ 25, <30 | 0.94 (0.91, 0.96) |  |
| ≥ 30 | 0.93 (0.89, 0.97) |  |
| Physical activity (MET-min/week) | | 0.837 |
| <500 | 0.95 (0.91, 1.00) |  |
| ≥ 500 | 0.94 (0.92, 0.96) |  |
| Missing data | 0.94 (0.91, 0.97) |  |
| Poor oral health |  | 0.997 |
| No | 0.94 (0.92, 0.96) |  |
| Yes | 0.94 (0.89, 0.99) |  |
| Missing data | 0.94 (0.90, 0.98) |  |
| Hypertension |  | 0.022 |
| No | 0.95 (0.93, 0.97) |  |
| Yes | 0.91 (0.88, 0.93) |  |
| Depression |  | 0.764 |
| No | 0.94 (0.92, 0.96) |  |
| Yes | 0.95 (0.91, 0.99) |  |
| Diabetes |  | 0.681 |
| No | 0.94 (0.92, 0.96) |  |
| Yes | 0.93 (0.89, 0.97) |  |
| Smoking status |  | 0.028 |
| Never | 0.94 (0.93, 0.96) |  |
| Former | 0.89 (0.86, 0.94) |  |
| Now | 0.96 (0.92, 1.00) |  |
| Drinking status |  | 0.036 |
| No | 0.97 (0.95, 0.99) |  |
| Yes | 0.93 (0.91, 0.96) |  |
| Milk |  | 0.687 |
| Often | 0.94 (0.91, 0.96) |  |
| Sometimes | 0.94 (0.90, 0.97) |  |
| Rarely | 0.91 (0.87, 0.96) |  |
| Never | 0.95 (0.91, 0.99) |  |
| Varied | 0.75 (0.49, 1.14) |  |
| Energy |  | 0.403 |
| T1 | 0.98 (0.93, 1.03) |  |
| T2 | 0.92(0.87,0.98) |  |
| T3 | 0.96 (0.92, 0.99) |  |
| Total fat |  | 0.595 |
| T1 | 0.96 (0.93, 1.00) |  |
| T2 | 0.94 (0.91, 0.97) |  |
| T3 | 0.96 (0.92, 1.00) |  |
| Dietary fiber |  | 0.757 |
| T1 | 0.95 (0.92, 0.98) |  |
| T2 | 0.96 (0.94, 0.99) |  |
| T3 | 0.95 (0.91, 0.99) |  |
| Selenium |  | 0.867 |
| T1 | 0.98 (0.94, 1.02) |  |
| T2 | 0.98 (0.93, 1.02) |  |
| T3 | 0.96 (0.93, 1.00) |  |
| Magnesium |  | 0.651 |
| T1 | 0.97 (0.92, 1.01) |  |
| T2 | 1.00 (0.95, 1.05) |  |
| T3 | 0.97 (0.93, 1.01) |  |
| Calcium |  | 0.593 |
| T1 | 0.91 (0.87, 0.96) |  |
| T2 | 0.93 (0.88, 0.98) |  |
| T3 | 0.94 (0.91, 0.98) |  |
| Sodium |  | 0.356 |
| T1 | 0.97 (0.93, 1.01) |  |
| T2 | 0.94 (0.90, 0.98) |  |
| T3 | 0.98 (0.95, 1.02) |  |
| Potassium |  | 0.591 |
| T1 | 0.96 (0.92, 1.01) |  |
| T2 | 0.93 (0.89, 0.98) |  |
| T3 | 0.97 (0.93, 1.00) |  |
| Plain water |  | 0.119 |
| T1 | 0.95 (0.92, 0.99) |  |
| T2 | 0.91 (0.88 0.94) |  |
| T3 | 0.94 (0.91, 0.97) |  |
| Missing data | 0.97 (0.93, 1.01) |  |
| Tap water |  | 0.197 |
| =0 | 0.94 (0.91, 0.98) |  |
| >0 | 0.93 (0.91, 0.95) |  |
| Missing data | 0.97 (0.93, 1.01) |  |
| Bottled water |  | 0.265 |
| =0 | 0.93 (0.91, 0.96) |  |
| >0 | 0.94 (0.91, 0.96) |  |
| Missing data | 0.97 (0.93, 1.01) |  |
| Tea |  | 0.098 |
| =0 | 0.94 (0.92, 0.97) |  |
| >0 | 0.92 (0.89, 0.94) |  |
| Missing data | 0.97 (0.93, 1.01) |  |
| Coffee |  | 0.222 |
| =0 | 0.93 (0.90, 0.96) |  |
| >0 | 0.94 (0.91, 0.97) |  |
| Missing data | 0.97 (0.93, 1.01) |  |

Note: the unit of dietary phosphorus in each layer is 0.1g.

**Supplementary Table 6.** Subgroup Analyses of the Association Between Phosphorus Intake and Constipation (stool frequency), weighted

| Characteristic | OR (95%CI) | P for interaction |
| --- | --- | --- |
| Sex |  | 0.352 |
| Female | 0.94 (0.89, 0.98) |  |
| Male | 0.90 (0.86, 0.95) |  |
| Age (yr) |  | 0.380 |
| <45 | 0.88 (0.84, 0.93) |  |
| ≥ 45,＜65 | 0.91 (0.88, 0.94) |  |
| ≥ 65 | 0.86 (0.79, 0.93) |  |
| Ethnicity |  | 0.014 |
| Non-Hispanic White | 0.86 (0.82, 0.91) |  |
| Mexican American | 0.90 (0.84, 0.96) |  |
| Non-Hispanic Black | 0.98 (0.94, 1.01) |  |
| Other Hispanic | 1.00 (0.91, 1.10) |  |
| Other Race | 0.89 (0.80, 0.99) |  |
| Education |  | 0.380 |
| < High school | 0.92 (0.87, 0.98) |  |
| High School | 0.86 (0.82, 0.92) |  |
| > High school | 0.90 (0.86, 0.95) |  |
| Income-poverty ratio (%) |  | 0.300 |
| <2 | 0.91 (0.88, 0.94) |  |
| ≥ 2 | 0.88 (0.82, 0.94) |  |
| Missing data | 0.95 (0.88, 1.02) |  |
| BMI (kg/m2) |  | 0.966 |
| <25 | 0.89 (0.83, 0.96) |  |
| ≥ 25, <30 | 0.88 (0.84, 0.93) |  |
| ≥ 30 | 0.89 (0.84, 0.93) |  |
| Physical activity (MET-min/week) | | 0.032 |
| <500 | 0.93 (0.90, 0.97) |  |
| ≥ 500 | 0.91 (0.87, 0.95) |  |
| Missing data | 0.85 (0.80, 0.89) |  |
| Poor oral health |  | 0.073 |
| No | 0.90 (0.86, 0.94) |  |
| Yes | 0.85 (0.78, 0.92) |  |
| Missing data | 0.93 (0.90, 0.96) |  |
| Hypertension |  | 0.847 |
| No | 0.89 (0.85, 0.93) |  |
| Yes | 0.89 (0.86, 0.91) |  |
| Depression |  | 0.038 |
| No | 0.89 (0.86, 0.91) |  |
| Yes | 0.95 (0.89, 1.02) |  |
| Diabetes |  | 0.453 |
| No | 0.89 (0.85, 0.92) |  |
| Yes | 0.92 (0.85, 0.98) |  |
| Smoking status |  | 0.132 |
| Never | 0.92 (0.89, 0.94) |  |
| Former | 0.89 (0.80, 0.99) |  |
| Now | 0.86 (0.81, 0.91) |  |
| Drinking status |  | 0.110 |
| No | 0.93 (0.89, 0.97) |  |
| Yes | 0.88 (0.84, 0.93) |  |
| Milk |  | 0.420 |
| Often | 0.92 (0.88, 0.95) |  |
| Sometimes | 0.88 (0.83, 0.92) |  |
| Rarely | 0.90 (0.80, 1.01) |  |
| Never | 0.88 (0.84, 0.94) |  |
| Varied | 0.64 (0.34, 1.23) |  |
| Energy |  | 0.788 |
| T1 | 0.87 (0.78, 0.98) |  |
| T2 | 0.86 (0.79, 0.93) |  |
| T3 | 0.89 (0.82, 0.97) |  |
| Total fat |  | 0.848 |
| T1 | 0.91 (0.80, 1.03) |  |
| T2 | 0.88 (0.84, 0.92) |  |
| T3 | 0.89 (0.84, 0.95) |  |
| Dietary fiber |  | 0.729 |
| T1 | 0.90 (0.85, 0.96) |  |
| T2 | 0.93 (0.89, 0.98) |  |
| T3 | 0.94 (0.87, 1.01) |  |
| Selenium |  | 0.822 |
| T1 | 0.91 (0.83, 1.00) |  |
| T2 | 0.88 (0.82, 0.94) |  |
| T3 | 0.90 (0.83, 0.98) |  |
| Magnesium |  | 0.163 |
| T1 | 0.94 (0.86, 1.02) |  |
| T2 | 1.00 (0.95, 1.04) |  |
| T3 | 0.93 (0.84, 1.03) |  |
| Calcium |  | 0.127 |
| T1 | 0.91 (0.84, 0.99) |  |
| T2 | 0.85 (0.79, 0.92) |  |
| T3 | 0.93 (0.87, 1.01) |  |
| Sodium |  | 0.908 |
| T1 | 0.89 (0.82, 0.97) |  |
| T2 | 0.91 (0.85, 0.98) |  |
| T3 | 0.89 (0.82, 0.97) |  |
| Potassium |  | 0.798 |
| T1 | 0.92 (0.84, 1.01) |  |
| T2 | 0.95 (0.90, 1.01) |  |
| T3 | 0.96 (0.88, 1.05) |  |
| Plain water |  | 0.004 |
| T1 | 0.89 (0.86, 0.93) |  |
| T2 | 0.87 (0.83, 0.93) |  |
| T3 | 0.86 (0.82, 0.89) |  |
| Missing data | 1.01 (0.92, 1.10) |  |
| Tap water |  | 0.003 |
| =0 | 0.86 (0.82, 0.90) |  |
| >0 | 0.89 (0.86, 0.93) |  |
| Missing data | 1.01 (0.92, 1.10) |  |
| Bottled water |  | 0.002 |
| =0 | 0.88 (0.85, 0.91) |  |
| >0 | 0.85 (0.82, 0.89) |  |
| Missing data | 1.01 (0.92, 1.10) |  |
| Tea |  | 0.002 |
| =0 | 0.87 (0.84, 0.90) |  |
| >0 | 0.88 (0.85, 0.92) |  |
| Missing data | 1.01 (0.92, 1.10) |  |
| Coffee |  | <0.001 |
| =0 | 0.91 (0.88, 0.94) |  |
| >0 | 0.83 (0.79, 0.87) |  |
| Missing data | 1.01 (0.92, 1.10) |  |

Note: the unit of dietary phosphorus in each layer is 0.1g.

**Supplementary Table 7.** Regression Analyses of the Association Between Dietary Phosphorus Intake and Chronic Constipation (stool consistency) from Post-imputation, weighted.

| Exposure | Model Ⅰ | Model Ⅱ | Model Ⅲ |
| --- | --- | --- | --- |
| Overall | 13948 | 13948 | 13948 |
| Phosphorus for each 0.1g | 0.94 (0.92,0.96) | 0.97 (0.96, 0.99) | 0.97 (0.95, 1.00) |
| Per 1 SD | 0.71 (0.64, 0.78) | 0.83(0.75,0.91) | 0.85(0.74,0.98) |
| Q1 | Ref. | Ref. | Ref. |
| Q2 | 0.92(0.70,1.20) | 1.02(0.79,1.33) | 1.08(0.81,1.43) |
| Q3 | 0.62(0.47,0.82) | 0.77(0.58,1.02) | 0.84(0.62,1.14) |
| Q4 | 0.46(0.36,0.59) | 0.70(0.55,0.89) | 0.79(0.53,1.16) |
| Men | 7028 | 7028 | 7028 |
| Phosphorus for each 0.1g | 0.95(0.92,0.98) | 0.96(0.92,0.99) | 0.96(0.90,1.01) |
| Per 1 SD | 0.73(0.60,0.90) | 0.76(0.63,0.93) | 0.77(0.55,1.08) |
| Q1 | Ref. | Ref. | Ref. |
| Q2 | 0.54(0.35,0.84) | 0.57(0.37,0.87) | 0.62(0.40,0.97) |
| Q3 | 0.59(0.38,0.90) | 0.62(0.40,0.97) | 0.69(0.42,1.13) |
| Q4 | 0.40(0.25,0.63) | 0.42(0.27,0.66) | 0.43(0.21,0.89) |
| Female | 6920 | 6920 | 6920 |
| Phosphorus for each 0.1g | 0.97(0.95,1.00) | 0.98(0.96,1.00) | 0.99(0.95,1.02) |
| Per 1 SD | 0.89(0.81,0.98) | 0.90(0.82,0.99) | 0.94(0.81,1.09) |
| Q1 | Ref. | Ref. | Ref. |
| Q2 | 1.12(0.83,1.50) | 1.14(0.85,1.54) | 1.19(0.88,1.60) |
| Q3 | 0.82(0.64,1.07) | 0.87(0.66,1.13) | 0.94(0.69,1.26) |
| Q4 | 0.80(0.61,1.06) | 0.83(0.63,1.11) | 0.92(0.63,1.36) |

Model Ⅰ was not adjusted.

Model Ⅱ was adjusted for age, sex and ethnicity. age (<45, ≥ 45＜65, and ≥ 65 years old), sex and ethnicity (Non-Hispanic White, Mexican American, Non-Hispanic Black, Other Hispanic, and Other Race).

Model Ⅲ was adjusted for adjusted for age (< 45, ≥ 45＜ 65, and ≥ 65 years old), sex and ethnicity (Non-Hispanic White, Mexican American, Non-Hispanic Black, Other Hispanic, and Other Race), BMI (< 25kg/m2, 25-29.9 kg/m2, and ≥ 30 kg/m2), Physical activity (MET-min/week) (< 500, ≥ 500), poor oral health (yes, no), hypertension (yes, no), depression (yes, no), diabetes (yes, no), smoking status (never, former, and now), drinking status (yes, no), milk (often, sometimes, rarely, never), energy (T1 < 1590.0; T2, 1590.0-2247.0; T3 ≥ 2247.1 kcal/day), and income-poverty ratio (%) (< 2, ≥ 2).

**Supplementary Table 8.** Regression Analyses of the Association Between Dietary Phosphorus Intake and Chronic Constipation (stool frequency) from Post-imputation, weighted.

| Exposure | Model Ⅰ | Model Ⅱ | Model Ⅲ |
| --- | --- | --- | --- |
| Overall | 13948 | 13948 | 13948 |
| Phosphorus for each 0.1g | 0.89(0.86,0.92) | 0.93(0.90,0.97) | 0.94(0.89,0.99) |
| Per 1 SD | 0.53(0.43,0.64) | 0.68(0.55,0.83) | 0.71(0.53,0.93) |
| Q1 | Ref. | Ref. | Ref. |
| Q2 | 0.71(0.53,0.95) | 1.02(0.79,1.33) | 0.88(0.65,1.20) |
| Q3 | 0.48(0.34,0.68) | 0.77(0.58,1.02) | 0.69(0.44,1.08) |
| Q4 | 0.20(0.12,0.32) | 0.70(0.55,0.89) | 0.38(0.19,0.76) |
| Men | 7028 | 7028 | 7028 |
| Phosphorus for each 0.1g | 0.90(0.86,0.95) | 0.92(0.88,0.97) | 0.91(0.86,0.97) |
| Per 1 SD | 0.54(0.40,0.74) | 0.61(0.45,0.84) | 0.58(0.41,0.82) |
| Q1 | Ref. | Ref. | Ref. |
| Q2 | 0.59(0.31,1.10) | 0.68(0.36,1.30) | 0.60(0.27,1.36) |
| Q3 | 0.26(0.14,0.50) | 0.33(0.17,0.63) | 0.25(0.10,0.62) |
| Q4 | 0.22(0.10,0.49) | 0.29(0.13,0.65) | 0.20(0.08,0.50) |
| Female | 6920 | 6920 | 6920 |
| Phosphorus for each 0.1g | 0.94(0.89,0.98) | 0.94(0.90,0.98) | 0.95(0.88,1.03) |
| Per 1 SD | 0.75(0.61,0.93) | 0.76(0.62,0.93) | 0.81(0.61,1.06) |
| Q1 | Ref. | Ref. | Ref. |
| Q2 | 0.63(0.42,0.94) | 0.66(0.44,0.98) | 0.62(0.39,0.96) |
| Q3 | 0.73(0.49,1.09) | 0.77(0.52,1.15) | 0.80(0.54,1.20) |
| Q4 | 0.47(0.31,0.71) | 0.47(0.31,0.72) | 0.58(0.33,1.03) |

Model Ⅰ was not adjusted.

Model Ⅱ was adjusted for age (<45, ≥ 45＜65, and ≥ 65 years old), sex and ethnicity (Non-Hispanic White, Mexican American, Non-Hispanic Black, Other Hispanic, and Other Race).

Model Ⅲ was adjusted for age (< 45, ≥ 45＜ 65, and ≥ 65 years old), sex and ethnicity (Non-Hispanic White, Mexican American, Non-Hispanic Black, Other Hispanic, and Other Race), BMI (< 25kg/m2, 25-29.9 kg/m2, and ≥ 30 kg/m2), Physical activity (MET-min/week) (< 500, ≥ 500), poor oral health (yes, no), hypertension (yes, no), depression (yes, no), diabetes (yes, no), smoking status (never, former, and now), drinking status (yes, no), milk (often, sometimes, rarely, never), energy (T1 < 1590.0; T2, 1590.0-2247.0; T3 ≥ 2247.1 kcal/day), and income-poverty ratio (%) (< 2, ≥ 2).
